# Colloidal Synthesis of Monodisperse High-Entropy Rare-Earth Oxide Nanoplatelets

**DOI:** 10.1021/acsnanoscienceau.6c00114

**Published:** 2026-09-04

**Authors:** Hanxin Fu, Lingchao Wang, Nachuan Li, Yi Zhou, Siyu Wang, Lianpeng Song, Yakun Yuan, Yifan Sun

**Affiliations:** † Frontiers Science Center for Transformative Molecules, State Key Laboratory of Synergistic Chem-Bio Synthesis, School of Chemistry and Chemical Engineering, and Zhangjiang Institute for Advanced Study, 12474 Shanghai Jiao Tong University, Shanghai 200240, China; ‡ Future Material Innovation Center, School of Materials Science and Engineering, Zhangjiang Institute for Advanced Study, 12474 Shanghai Jiao Tong University, Shanghai 200240, China

**Keywords:** high-entropy oxides, rare-earth oxides, colloidal synthesis, short-range order, two-dimensional nanomaterials

## Abstract

High-entropy rare-earth oxides (HE-REOs) offer a broad compositional space for tuning defect chemistry, lattice distortion, and local atomic environments, yet their controlled synthesis as monodisperse and structurally well-defined nanostructures remains challenging. Here, we report a colloidal approach for the synthesis of solution-dispersible, single-crystalline HE-REO nanoplatelets. The ultrathin two-dimensional nanoplatelets adopt a trigonal REO lattice that supports homogeneous multication incorporation across ternary to denary HE-REO compositions, and a simultaneous multication nucleation and growth pathway is demonstrated. Atomic-resolution imaging, combined with binary-type intensity classification, enables assessment of local chemical ordering, and increasing configurational entropy drives the system from a nearly random local structure toward a heteroelement-dominated short-range ordered state. High-entropy design further facilitates the incorporation of chemically dissimilar nonrare-earth cations, expanding the accessible miscibility window of REO nanostructures. Our work establishes colloidal synthesis as both a versatile route to monodisperse high-entropy oxide nanostructures and a model platform for probing and understanding entropy-governed behavior and functionality in high-entropy materials.

## Graphical abstract

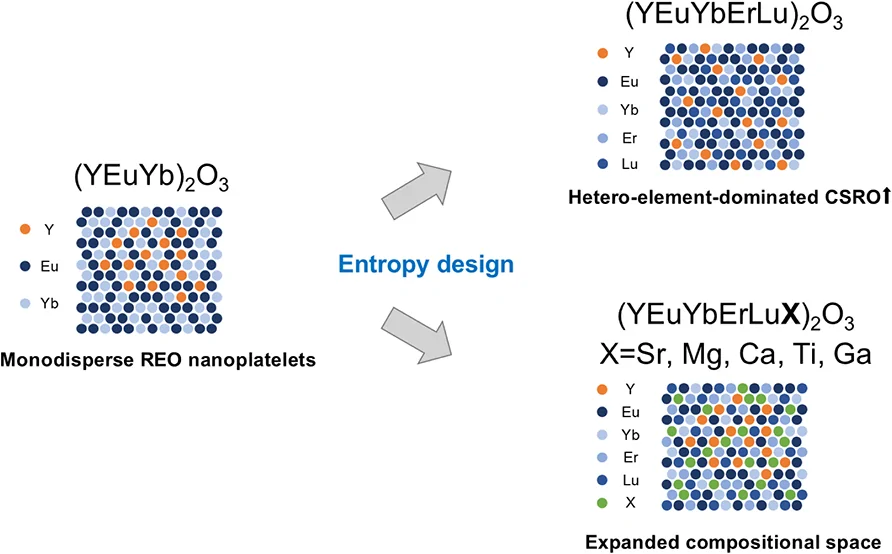

## Introduction

High-entropy oxides (HEOs), in which typically five or more metal cations are incorporated into a single crystalline lattice, have emerged as a versatile class of complex oxides with an exceptionally broad compositional and structural design space.^1,2^ Among them, high-entropy rare-earth oxides (HE-REOs) represent an important family because rare-earth cations generally share similar valence states, ionic sizes and coordination preferences. This chemical compatibility allows multiple lanthanide and lanthanide-like cations, such as Y^3+^, to be accommodated within a common oxide framework with relatively low enthalpic penalties, providing an ideal platform for systematically tuning configurational entropy while maintaining lattice coherence.^3–5^ Beyond compositional diversity, the disordered distribution of rare-earth cations can generate lattice distortion, local strain and heterogeneous metal–oxygen coordination environments, which may modulate oxygen-vacancy formation, oxygen mobility and lattice-oxygen activity.^6–8^ These features make HE-REOs attractive for heterogeneous catalysis,^9–12^ energy storage and conversion,^13–15^ ionic conduction,^16^ photonic materials and magnetism,^17,18^ where defect chemistry, redox tunability and local structural disorder play central roles in determining performance.

Despite these advantages, the controlled synthesis of HE-REOs with well-defined structures remains challenging, especially at the nanoscale. Conventionally, HE-REOs are prepared through high-temperature solid-state reactions, which promote cation interdiffusion and amplify the entropic contribution through the TΔS term to overcome the enthalpic penalty associated with multicomponent mixing.^19–22^ However, such high-temperature conditions usually drive the system toward thermodynamic equilibrium, yielding products dominated by stable bulk phases and making it difficult to control nanoscale structural parameters, such as particle size, morphology and exposed facets. Solution-based nonequilibrium methods, such as sol–gel,^23^ coprecipitation and solution combustion approaches,^17,24^ have been developed to improve precursor homogeneity and reduce particle size, but subsequent high-temperature annealing is still required. The resulting nanoparticles often suffer from aggregation and morphological heterogeneity, which complicates the decoupling of intrinsic nanoscale solid-solution behavior from surface, interface and grain-boundary effects. This synthetic limitation also restricts the investigation of local atomic arrangements, such as chemical short-range order (CSRO), whose subtle deviations from ideal cation disorder can influence enthalpy–entropy compensation, phase stability and functional properties.^25–28^ Although local chemical ordering has been extensively studied in metallic high-entropy alloys (HEAs), its observation and interpretation in HEOs, particularly HE-REOs, remain challenging due to the lack of well-defined nanoscale model systems.^26,27,29,30^

Colloidal synthesis offers a unique route to addressing these challenges.^31,32^ First, unlike solid-state routes, solution-phase reactions allow different metal precursors to be homogeneously mixed at the molecular level before crystallization, reducing long-range diffusion barriers under mild conditions.^33^ Second, molecular ligands can regulate and balance the distinct reactivities of different metal species. For example, recent colloidal synthesis of high-entropy spinel oxide nanocrystals showed that ligand-mediated control of esterification kinetics can equalize cation monomer production and produce highly monodisperse HEO nanocrystals.^34^ Third, ligand-directed nucleation and growth enable precise control over the size, dimensionality, and phase structure, while surface-bound ligands can effectively suppress aggregation.^35,36^ These favorable traits make colloidal synthesis particularly suitable for constructing solution-dispersible HEO nanocrystals as structurally well-defined building blocks.

Herein, we report a colloidal synthetic protocol for monodisperse HE-REO nanoplatelets. The resulting ultrathin two-dimensional (2D) nanoplatelets adopt a trigonal REO structure and enable multication incorporation into a single-phase oxide lattice under mild solution-phase conditions. This colloidal approach is applicable to a family of ternary, quinary, octonary and denary HE-REO nanoplatelets, allowing systematic control over composition and configurational entropy. Atomic-resolution microscopic imaging combined with binary-type Z-contrast classification, further reveals that increasing configurational entropy drives the local structure from a nearly random configuration toward a heteroelement-dominated short-range ordered state. Aliquot studies reveal a simultaneous formation pathway, in which the designed stoichiometry is established at the earliest nucleation stage and preserved during subsequent growth. In addition, high-entropy design allows the incorporation of otherwise immiscible cations, expanding the accessible compositional space. This work establishes colloidal synthesis as a versatile platform for designing monodisperse HE-REO nanostructures with tunable compositions and local atomic structures.

## Experimental Section

### Chemicals and Materials

Yttrium­(III) acetate tetrahydrate [Y­(CH_3_COO)_3_·4H_2_O, 99.99%] and terbium­(III) nitrate hexahydrate [Tb­(NO_3_)_3_·6H_2_O, 99.99%] were purchased from Meryer (Shanghai) Chemical Technology Co., Ltd. Europium­(III) acetylacetonate hydrate [Eu­(acac)_3_·*x*H_2_O, ≥99%] was purchased from Shanghai Yuanye Bio-Technology Co., Ltd. Gadolinium­(III) acetate hydrate [Gd­(CH_3_COO)_3_·*x*H_2_O, 99.9%] and cerium­(III) acetate hydrate [Ce­(CH_3_COO)_3_·*x*H_2_O, 99.9%] were purchased from InnoChem Technology Co., Ltd. Terbium­(III) acetate hexahydrate [Tb­(CH_3_COO)_3_·6H_2_O, 99.9%], holmium­(III) acetate hexahydrate [Ho­(CH_3_COO)_3_·*x*H_2_O, 99.9%], erbium­(III) acetate hydrate [Er­(CH_3_COO)_3_·*x*H_2_O, 99.9%], europium­(III) nitrate [Eu­(NO_3_)_3_, ≥99.99%], and calcium acetate hydrate [Ca­(CH_3_COO)_2_·*x*H_2_O, 99.995%] were purchased from Shanghai Macklin Biochemical Co., Ltd. Dysprosium­(III) acetate hexahydrate [Dy­(CH_3_COO)_3_·6H_2_O, 99.9%] and strontium acetate hemihydrate [Sr­(CH_3_COO)_2_·0.5H_2_O, 99%] were purchased from Shanghai Xushuo Biotechnology Co., Ltd. Thulium­(III) acetate hydrate [Tm­(CH_3_COO)_3_·*x*H_2_O, ≥99%] and ytterbium­(III) acetate hydrate [Yb­(CH_3_COO)_3_·*x*H_2_O, 99.99%] were purchased from Leyan Scientific Co., Ltd. Lutetium­(III) acetate [Lu­(CH_3_COO)_3_·H_2_O, 99.99%], cerium­(III) nitrate hexahydrate [Ce­(NO_3_)_3_·6H_2_O, 99.99%], thulium­(III) nitrate [Tm­(NO_3_)_3_·5H_2_O, 99.99%] and magnesium acetate tetrahydrate [Mg­(CH_3_COO)_2_·4H_2_O, 98%] were purchased from Shanghai Adamas Reagent Co., Ltd. Holmium­(III) nitrate pentahydrate [Ho­(NO_3_)_3_·5H_2_O, 99.9%], titanium­(IV) oxide acetylacetonate (C_10_H_14_O_5_Ti, 98%) and gallium nitrate hydrate [Ga­(NO_3_)_3_·*x*H_2_O, 99.99%] were purchased from Aladdin. Oleic acid (OA, technical grade, 90%) was purchased from Alfa Aesar. Oleylamine (OAm, technical grade, 80–90%] and 1-octadecene [ODE, technical grade, 90%] were purchased from Sigma-Aldrich. Solvents, including hexane, ethanol and acetone, were of analytical grade. All chemicals were used as received without further purification.

### Colloidal Synthesis of Eu_2_O_3_ and (CeEuHoTbTm)_2_O_3_ 2D Nanoplatelets

All reactions were carried out under a nitrogen atmosphere using standard Schlenk techniques and workup procedures were performed in air. Eu_2_O_3_ nanoplatelets were synthesized via a colloidal thermal decomposition method.^37^ In a typical synthesis, europium­(III) acetylacetonate [Eu­(acac)_3_·*x*H_2_O, 0.4 mmol], oleic acid (OA, 90%, 11.25 mL), and oleylamine (OAm, 80–90%, 18.75 mL) were added to a 100 mL three-neck round-bottom, degassed for 30 min under vacuum at 100 °C and then switched to nitrogen. The reaction mixture was further heated to 300 °C under nitrogen atmosphere and maintained for 1 h. After naturally cooling to room temperature, the products were washed three times with a 1:1 hexane/ethanol mixture and redispersed in hexane for further characterization. The (CeEuHoTbTm)_2_O_3_ nanoplatelets with trigonal structure were fabricated using the same protocol except replacing europium­(III) acetylacetonate with equal moles of cerium­(III) acetate hydrate [Ce­(CH_3_COO)_3_·*x*H_2_O], europium­(III) acetylacetonate hydrate [Eu­(acac)_3_·*x*H_2_O], holmium­(III) acetate hydrate [Ho­(CH_3_COO)_3_·*x*H_2_O], terbium­(III) acetate hydrate [Tb­(CH_3_COO)_3_·6H_2_O], and thulium­(III) acetate hydrate [Tm­(CH_3_COO)_3_·*x*H_2_O] (0.18 mmol each), and elevating the reaction temperature from 300 to 340 °C. Ternary [(YEuYb)_2_O_3_], quinary [(YEuYbErLu)_2_O_3_], octonary [(YEuYbErLuDyHoTm)_2_O_3_], and denary [(YEuYbErLuDyHoTmGdTb)_2_O_3_] samples were prepared using the same method to adjust configurational entropy, and the total metal precursor amount was set as 0.9 mmol for consistency. For quantitative chemical short-range order (CSRO) analysis, where the Y content was fixed at 10 at.% and the configurational entropy was varied by increasing the number of rare-earth species, an additional series consisting of binary [(Y_0.1_Eu_0.9_)_2_O_3_], ternary [(Y_0.1_Eu_0.45_Yb_0.45_)_2_O_3_], and quinary [(Y_0.1_Eu_0.225_Yb_0.225_Er_0.225_Lu_0.225_)_2_O_3_] compositions was synthesized under identical reaction conditions. Additional elements including Mg, Ca, Ti, and Ga were further introduced to the synthesis procedure to examine the compositional flexibility of the trigonal HE-REO framework. These nonrare-earth metal incorporation experiments were conducted using the same colloidal synthesis protocol established for HE-REO nanoplatelets. The corresponding nonrare-earth metal precursors were added by partially replacing the rare-earth metal precursors while maintaining the designed total metal precursor amount. All other synthesis parameters were kept identical.

### Colloidal Synthesis of (CeEuHoTbTm)_2_O_3_ 3D Nanoparticles

HE-REO nanoparticles with thermodynamically stable 3D structures were prepared based on the reported protocol for colloidal CeO_2_ nanoparticles.^38^ In a typical synthesis, cerium­(III) nitrate hexahydrate [Ce­(NO_3_)_3_·6H_2_O], europium­(III) nitrate hexahydrate [Eu­(NO_3_)_3_·6H_2_O], holmium­(III) nitrate pentahydrate [Ho­(NO_3_)_3_·5H_2_O], terbium­(III) nitrate hexahydrate [Tb­(NO_3_)_3_·6H_2_O], and thulium­(III) nitrate pentahydrate [Tm­(NO_3_)_3_·5H_2_O] (0.4 mmol each) were dissolved in 1-octadecene (ODE, 13 mL) and oleylamine (OAm, 2 mL). The mixture was degassed and purged with nitrogen three times, heated to 80 °C for 30 min, and then further heated to 250 °C for 2 h under nitrogen. After cooling, the product was washed with a 1:1 acetone/ethanol mixture and redispersed in hexane for further characterization.

### Thermal Treatment of HE-REO Nanostructures

To evaluate the thermal stability of the metastable trigonal structure, Eu_2_O_3_ 2D nanoplatelets were thermally annealed in a muffle furnace under air. The samples were heated to either 400 or 700 °C at a ramping rate of 5 °C/min and maintained at the target temperature for 5 h, and then naturally cooled to room temperature. Thermal annealing was also performed to investigate the formation of HE-REOs with thermodynamically stable phases. The 3D (CeEuHoTbTm)_2_O_3_ nanoparticles obtained after colloidal synthesis consist of mixed precursor phases rather than a single crystallographic phase. To promote single-phase HE-REO formation, the as-synthesized nanoparticle samples were thermally annealed in air at 300 °C for 3 h. For comparison, the metastable 2D nanoplatelets were subjected to the same thermal treatment under identical conditions, which enables a direct comparison of phase evolution and cation-distribution behavior between the stable and metastable phase structures.

### Characterization

Powder X-ray diffraction (XRD) patterns were recorded on a Thermo Scientific ARL EQUINOX 3000 diffractometer using Cu Kα radiation (λ = 1.5406 Å). Low-magnification and high-resolution high-angle annular dark-field scanning transmission electron microscopy (HAADF-STEM) images, transmission electron microscopy (TEM) images, together with energy-dispersive X-ray spectroscopy (EDS) elemental maps, were acquired using a Thermo Scientific Talos F200X G2 transmission electron microscope operated at 200 kV. For sample preparation, a drop of dilute nanoparticle dispersion was cast onto a carbon-coated copper (Cu) TEM grid and dried under ambient conditions. Elemental mapping and compositional analysis were performed using element-specific characteristic X-ray lines. For light elements such as Y, Sr, Mg, Ca, Ti, and Ga, K-shell emission lines were used for quantification, whereas for rare-earth elements including Ce, Eu, Ho, Tb, Tm, Er, Yb, Lu, Dy, and Gd, L-shell emission lines were used. The principal characteristic X-ray lines and their corresponding peak energies are summarized in Tables S1 and S2. All EDS data were processed using Thermo Scientific Velox software. Peak fitting and deconvolution of overlapping characteristic X-ray lines were automatically performed using the built-in fitting routine based on the Brown–Powell ionization cross-section model together with multipolynomial background correction. Elemental quantification was performed based on single representative EDS maps collected from regions containing multiple nanoplatelets or nanoparticles to ensure sufficient signal collection for quantitative analyses. The resulting compositions were used to assess consistency with the nominal stoichiometry. Because relatively uniform elemental distributions were observed within each field of view, statistical averaging over multiple independent maps was not performed. Atomic-resolution HAADF-STEM images were acquired on an aberration-corrected transmission electron microscope (Thermo Fisher Scientific, Spectra 300) operated at 300 kV. The resulting Z-contrast images were used to examine local atomic arrangements, lattice structures and cation distributions at the nanoscale. Inductively coupled plasma optical emission spectroscopy (ICP-OES, Agilent 730) was used to determine the elemental composition of the samples.

### Local Chemical Ordering Analysis

The atomic positions were identified from the filtered STEM images by locating the local intensity maxima. The integrated intensity around each atomic column was extracted and used for atom classification. Based on the intensity distribution, the atomic columns were grouped into two different atomic types corresponding to Y and the rare-earth elements. To quantify the local chemical ordering behavior, chemical short-range order (CSRO) parameters (α*_ij_*) were calculated for each atomic site based on the nearest-neighbor configuration using the following equation $\alpha_{ij}=\frac{p_{ij}-C_{j}}{\delta_{ij}-C_{j}}$ , where *P_ij_* is the probability of finding a *j*-type atom among the nearest neighbors of an *i*-type atom, *Cj* represents the concentration of *j*-type atoms in the sample, and *δ*_*ij*_ is the Kronecker delta function.^39,40^

## Results and Discussion

### Synthetic Extension from Eu_2_O_3_ to HE-REO Nanoplatelets

Controlling multication incorporation in oxide systems requires a host lattice capable of accommodating chemical complexity without imposing a strong energetic driving force for phase segregation. REOs provide a suitable platform in this regard because rare-earth cations generally share similar valence states, coordination preferences and chemical characteristics, while their ionic radii remain relatively close across the lanthanide series owing to lanthanide contraction.^41,42^ This chemical compatibility can reduce the enthalpic penalty associated with multication mixing, allowing configurational entropy to play a decisive role in stabilizing HEO lattices. In addition, phase metastability offers a kinetic handle for promoting homogeneous multication incorporation, which is crucial for low-temperature solution-phase synthesis. Unlike thermodynamically stable phases, metastable lattices crystallize under nonequilibrium conditions, allowing homogeneously mixed cations to be incorporated into a nascent oxide lattice before long-range diffusion and compositional segregation occur. Consistent with the Ostwald’s step rule, such metastable crystallization can kinetically preserve early stage local chemical environments and lower the barrier for multicomponent solid-solution formation. In colloidal systems, this kinetic pathway can be further regulated by molecular ligands, which modulate monomer generation, surface passivation and facet-selective growth.

Guided by the above rationale, we selected Eu_2_O_3_ as a model platform, because of its rich structural polymorphism attained in colloidal synthesis, for the synthesis of HE-REOs. As shown in Figure S1a–S1c, Eu_2_O_3_ can exist as a thermodynamically stable cubic phase and as monoclinic or trigonal polymorphs that are metastable under ambient conditions.^43^ Notably, trigonal REO phases are typically obtained only under extreme conditions, such as 6 GPa pressures or temperatures above 1000–2000 °C.^44–46^ In contrast, low-temperature colloidal synthesis enables the formation of a trigonal Eu_2_O_3_ lattice under mild conditions,^47^ suggesting that nonequilibrium crystallization can kinetically stabilize metastable REO polymorphs. Moreover, this metastable phase is structurally compatible with a wide range of REOs.^41^ This compatibility enables different rare-earth elements to be incorporated into a shared metastable framework, while the intrinsic tolerance of REO lattices to distortion and oxygen-defect formation further supports multication accommodation. Thus, the metastable REO phase provides a low-temperature solution-phase platform for constructing HE-REO nanostructures.

Colloidal Eu_2_O_3_ nanoplatelets were synthesized using a reported protocol,^37^ as detailed in the Experimental Section. As shown in Figure 1a, the XRD pattern of the as-synthesized Eu_2_O_3_ nanoplatelets exhibits two broad diffraction features, which can be attributed to the overlapping (100), (002), and (11̅1) reflections and the (12̅0) reflection of the metastable trigonal phase. This diffraction profile is observably different from the simulated patterns of cubic and monoclinic Eu_2_O_3_, supporting the formation of trigonal Eu_2_O_3_.^44^ TEM images (Figure 1b) reveal a well-defined 2D disk-like morphology with ultrathin thicknesses, as further confirmed by the side-view images. Size-distribution analysis gives an average diameter of 14.4 ± 2.3 nm, supporting the high monodispersity achieved by colloidal synthesis. Atomic resolution HAADF-STEM imaging (Figure 1c) shows continuous lattice contrast across an individual nanoplatelet, indicating its single-crystalline nature. The corresponding fast Fourier transform (FFT) pattern exhibits a hexagonal arrangement of diffraction spots, consistent with the *c*-axis projection of a trigonal lattice with intrinsic 3-fold symmetry. Although cubic Eu_2_O_3_ viewed along the [111] zone axis can also produce a hexagonal FFT pattern, quantitative comparison of the reciprocal lattice spacings distinguishes it from the trigonal structure (Figures S1 and S2).

**Figure 1:**
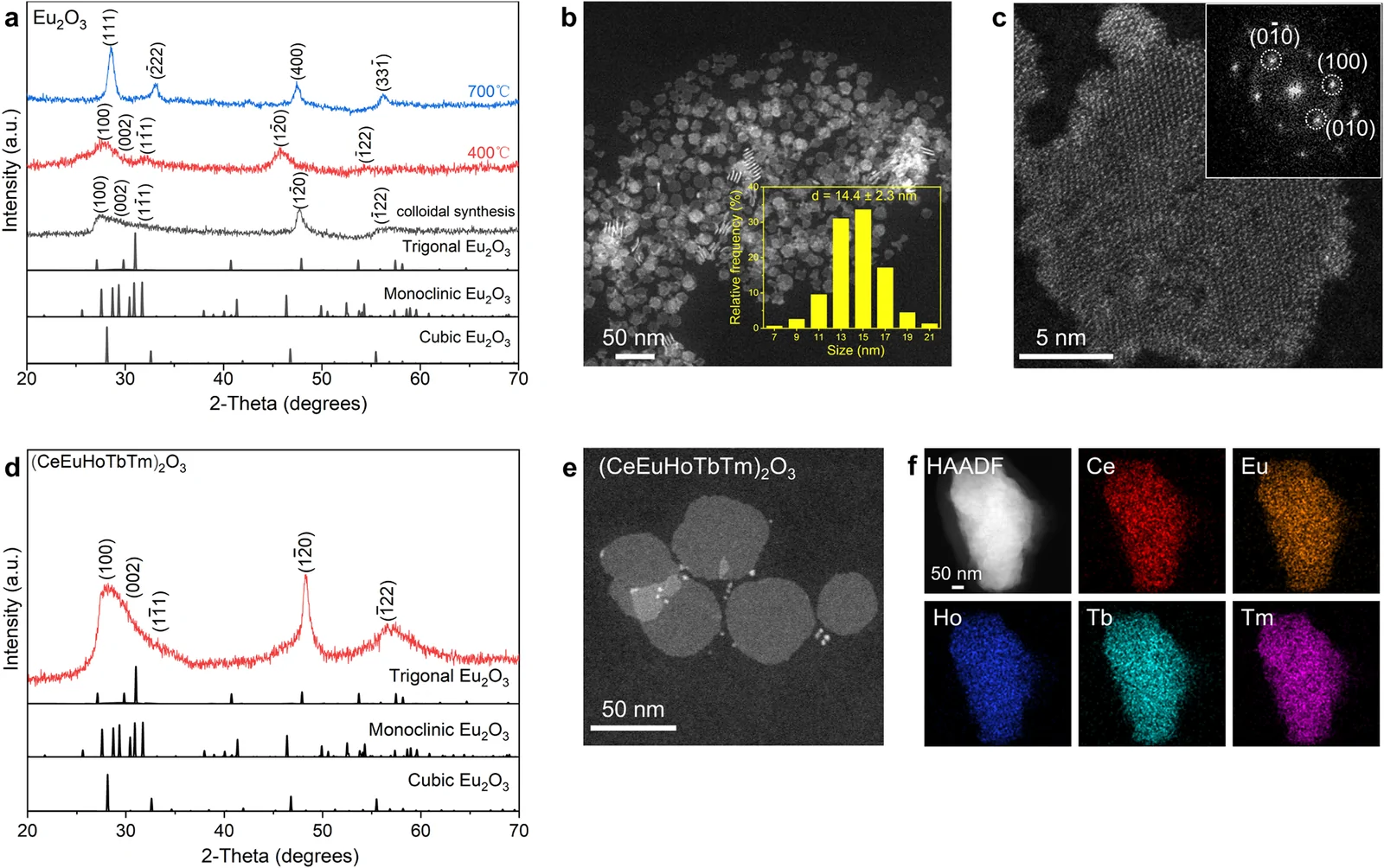
Characterization of colloidal Eu_2_O_3_ and (CeEuHoTbTm)_2_O_3_ nanoplatelets. (a) XRD patterns of Eu_2_O_3_ nanoplatelets before and after thermal annealing at different temperatures, as well as simulated patterns of bulk trigonal, monoclinic and cubic Eu_2_O_3_ for comparison. (b) HAADF-STEM image and lateral size distribution histogram of colloidal Eu_2_O_3_ nanoplatelets. (c) Atomic-resolution HAADF-STEM image of colloidal Eu_2_O_3_ nanoplatelets, with the corresponding FFT pattern shown in the inset. (d) XRD pattern of colloidal (CeEuHoTbTm)_2_O_3_ nanoplatelets, with simulated patterns of bulk trigonal, monoclinic and cubic Eu_2_O_3_ for comparison. (e) HAADF-STEM image and (f) STEM-EDS elemental maps of the colloidal (CeEuHoTbTm)_2_O_3_ nanoplatelets.

Thermal annealing was conducted to investigate the phase stability of the as-synthesized trigonal Eu_2_O_3_ nanoplatelets. After annealing at 400 °C in air, the overall XRD profile is largely preserved (Figure 1a). Notably, the (12̅0) reflection displays a subtle shift toward lower diffraction angles, suggesting mild structural relaxation rather than complete phase transformation. The corresponding FFT pattern also preserves the characteristic hexagonal symmetry (Figure S3). A few weak additional diffraction spots are also observed, which may arise from overlap and stacking of the nanoplatelets upon annealing. In contrast, annealing at 700 °C in air gives rise to a clear phase conversion to the thermodynamically stable cubic Eu_2_O_3_ phase based on the indexed diffraction pattern. This thermally induced trigonal-to-cubic phase transition further confirms the metastable nature of the colloidally synthesized Eu_2_O_3_ nanoplatelets.

Using the same protocol, we next interrogated the synthesis of HE-REOs with the composition of (CeEuHoTbTm)_2_O_3_. As shown in Figure 1d (Figure S4), the XRD pattern of the as-synthesized product exhibits diffraction features similar to those of metastable trigonal Eu_2_O_3_ nanoplatelets, indicating that multication incorporation preserves the underlying trigonal lattice, with no detectable impurity peaks or secondary phases, and leads to a single-phase HE-REO structure. TEM images and STEM-EDS elemental maps further confirm the retained disk-like morphology and homogeneous distribution of Ce, Eu, Ho, Tb and Tm throughout the nanoplatelets (Figure 1e–f). The preservation of the ultrathin disk-like morphology suggests that multication incorporation does not disrupt the ligand-mediated anisotropic growth pathway. In the oleic acid/oleylamine system, oleate ligands generated through acid–base interactions can selectively passivate the basal surfaces, suppressing out-of-plane growth while allowing lateral growth along the nanoplatelet edges. This result indicates that introducing multiple rare-earth cations does not create obvious grain boundaries or compromise the crystallographic coherence of the metastable trigonal lattice. Quantitative STEM-EDS analysis shows that the overall composition remains close to the nominal stoichiometry (Figure S5). Compared with the unary Eu_2_O_3_ nanoplatelets, the high-entropy sample exhibits a slightly increased lateral size. This difference is likely associated with the altered nucleation and growth kinetics in the multicomponent system, where different metal precursors can exhibit different coordination reactivities and monomer release rates, and interactions with oleic acid and oleylamine may alter facet-selective growth and lead to changes in lateral dimensions. The capability to directly access solution dispersible and monodisperse HE-REO nanoplatelets highlights the advantage of ligand-regulated colloidal chemistry. Surface-bound ligands suppress aggregation through steric stabilization, thereby overcoming the aggregation and morphological heterogeneity associated with thermally derived HE-REO nanopowders. Further ligand-exchange treatment enables dispersion of these nanoplatelets in diverse solvent systems and provides a handle for ligand-directed self-assembly into high-entropy superstructures. This colloidal processability opens opportunities for solution-processed HEO films, catalytic interfaces, bioactive architectures and rare-earth-based optical materials.

Note that a stable-phase colloidal route based on cubic CeO_2_ nanoparticles does not produce single-phase HE-REO nanostructures with the same nominal composition, as evidenced by the mixed diffraction patterns of EuO­(OH) and Eu_6_O­(NO_3_)_8_(OH)_8_ (Figure S6) and heterogeneities in morphologies (nanorods, irregular nanoplatelets and nanoscale clusters) and elemental distribution (Figure S7). To distinguish incomplete precursor conversion from intrinsic cation redistribution in the stable oxide lattice, the mixed-phase products were further annealed at 300 °C in air, yielding a crystallographically single cubic bixbyite phase (Figure S6). However, HAADF-STEM elemental mapping and line-scan profiles reveal pronounced nanoscale cation segregation, with Ce/Eu and Ho/Tb/Tm elements separated from each other, despite an overall composition close to the nominal ratio (Figure S8a–c). In contrast, metastable HE-REO nanoplatelets subjected to the same annealing treatment retain the uniform distribution of all five rare-earth cations (Figure S8d–f).

Collectively, these results indicate that the metastable trigonal Eu_2_O_3_-derived lattice provides a kinetically favorable platform for forming HE-REO nanostructures under low-temperature colloidal conditions. This behavior is consistent with previous studies showing that metastable crystalline phases often form when atomic diffusion is insufficient for the system to reach thermodynamic equilibrium, allowing nonequilibrium configurations to be preserved and elemental segregation to be suppressed.^48–50^ Importantly, the trigonal symmetry in this system should not be viewed as being directly selected by configurational entropy alone. Unlike conventional bulk high-entropy materials synthesized under near-equilibrium conditions, where entropy is often associated with high-symmetry phases, phase selection in these ultrathin colloidal nanoplatelets is strongly influenced by kinetic crystallization, surface-energy contributions and ligand-mediated stabilization. Thus, nonequilibrium colloidal growth and ligand passivation primarily favor retention of the metastable trigonal framework, whereas configurational entropy facilitates homogeneous multication incorporation within this framework and suppresses cation segregation. Therefore, the observed HE-REO formation is resulted by a combined effect of lattice metastability, colloidal ligand chemistry and chemical compatibility among rare-earth elements. These well-defined nanoplatelets provide a useful platform for understanding the formation mechanism, enthalpy–entropy compensation and local atomic ordering in HEOs.

### Compositional Design of HE-REO Nanoplatelets

Building on the established synthetic protocol for the (CeEuHoTbTm)_2_O_3_ nanoplatelets, we next explored the compositional tunability and generality of this platform. A series of single-phase HE-REO nanoplatelets with increasing compositional complexity were successfully prepared, including ternary (YEuYb)_2_O_3_, quinary (YEuYbErLu)_2_O_3_, octonary (YEuYbErLuDyHoTm)_2_O_3_ and denary (YEuYbErLuDyHoTmGdTb)_2_O_3_. The XRD patterns show that all compositions retain the characteristic diffraction features of the trigonal REO lattice (Figures 2a and S9). Notably, the reflection (12̅0) position gradually shifts toward higher diffraction angles with increasing component number. This trend is consistent with the decrease in the calculated average ionic radius of the incorporated cations (Figure 2b and Tables S3 and S4). No impurity peaks or secondary phases are observed within the detection limit of XRD, suggesting that the trigonal REO framework can accommodate increasing compositional complexity without apparent phase separation. The lattice spacings obtained from iFFT and XRD show consistent compositional evolution, indicating good agreement between local lattice measurements from individual nanoplatelets and ensemble-averaged structural information from XRD (Table S5).

**Figure 2:**
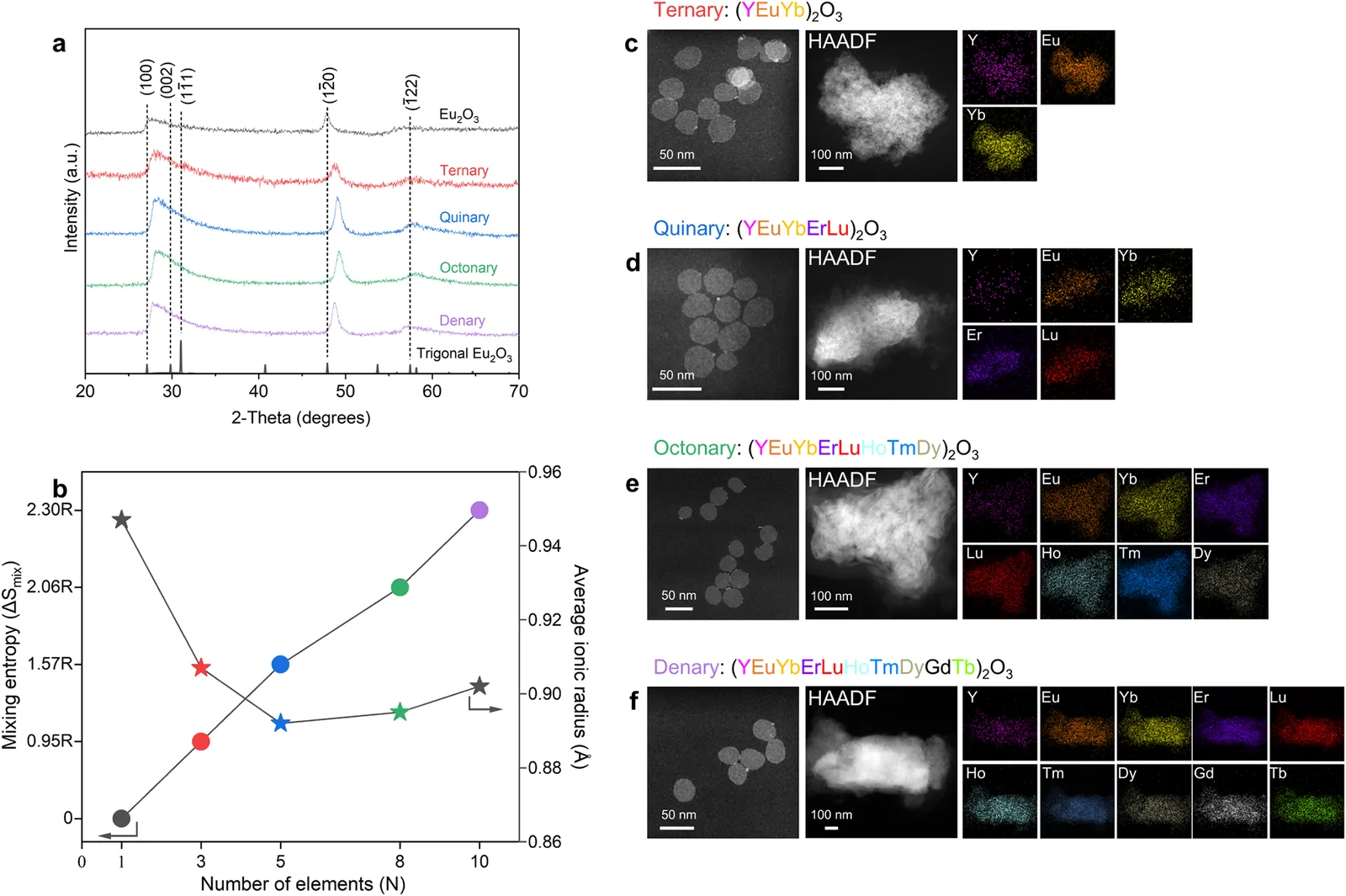
Compositional tunability of colloidal HE-REO nanoplatelets. (a) XRD patterns of colloidal Eu_2_O_3_, ternary, quinary, octonary, and denary HE-REO nanoplatelets. (b) Variation of configurational entropy and average ionic radius as a function of element number. HAADF-STEM images and corresponding STEM-EDS elemental maps of (c) ternary, (d) quinary, (e) octonary, and (f) denary HE-REO nanoplatelets.

HAADF-STEM imaging and corresponding STEM-EDS elemental maps further show that all compositions preserve the 2D disk-like morphology with relatively uniform size and shape (Figure 2c–f), and the thickness remains essentially unchanged (Figures S10). The constituent elements are distributed throughout the nanoplatelets without obvious macroscopic segregation, and the measured elemental ratios from EDS and ICP analyses are generally consistent with the designed stoichiometries (Figures S11 and S12). Minor deviations between EDS and ICP results (Figure S13) likely arise from the limited quantification accuracy of EDS for elements with overlapping spectral features and relatively weak local signals due to the ultrathin thickness of the HE-REO nanoplatelets. Together with the systematic increase in configurational entropy with increasing component number (Figure 2b), these results demonstrate the broad applicability of this colloidal method for incorporating diverse rare-earth cations into a shared oxide lattice while preserving the nanoplatelet morphology.

### Binary-Type Local Chemical Ordering in HE-REO Nanoplatelets

Building on the established HE-REO nanoplatelet platform with tunable compositions and well-defined structures, we next investigated how configurational entropy influences local chemical ordering at the nanoscale. Chemical short-range order (CSRO) is an important structural descriptor in high-entropy materials because subtle deviations from a fully random cation distribution can affect local strain, defect formation, phase stability and functional properties. However, direct identification of chemical ordering in HE-REOs is intrinsically challenging because the constituent rare-earth elements possess relatively similar atomic numbers, resulting in limited Z-contrast in HAADF-STEM imaging. Benefiting from the ultrathin 2D geometry of the nanoplatelets (Figures S14 and S15, Table S6), overlap from multiple atomic layers along the projection direction is minimized, which facilitates atomic-column intensity analysis. To enable quantitative analysis of local atomic configurations, we therefore simplified the HE-REO lattice into a binary-type representation based on atomic intensity contrast. Relatively weak atomic columns associated with Y^3+^ cations were assigned as type-1 columns, whereas stronger columns contributed by heavier rare-earth cations were grouped as type-2 columns.^51^ Accordingly, the CSRO analysis in this section should be interpreted as a binary-type analysis of Y–Y, Y–RE and grouped RE–RE correlations, rather than an element-specific description of all possible lanthanide–lanthanide pair correlations.

Specifically, we prepared binary (Y_0.1_Eu_0.9_)_2_O_3_, ternary (Y_0.1_Eu_0.45_Yb_0.45_)_2_O_3_, and quinary (Y_0.1_Eu_0.225_Er_0.225_Yb_0.225_Lu_0.225_)_2_O_3_ HE-REO nanoplatelets, in which the Y content was fixed at 10%. This compositional design maintains the relative population of type-1 and type-2 columns at approximately 1:9, which decouples the effect of compositional complexity from the relative abundance of the two atomic-column types (Figures S16 and S17). This allows us to evaluate how increasing compositional diversity within the grouped type-2 population influences Y-centered binary CSRO. A representative aberration-corrected HAADF-STEM image of the quinary (Y_0.1_Eu_0.225_Er_0.225_Yb_0.225_Lu_0.225_)_2_O_3_ HE-REO nanoplatelets shows resolved atomic columns (Figure 3a), where the darker atomic columns correspond to type 1 sites, while the brighter columns correspond to type 2 sites (Figure 3b). Statistical analysis of the identified atomic columns yields approximately 225 type 1 atoms and 2026 type 2 atoms (Figure 3c–d), consistent with the designed compositional ratio of approximately 1:9.

**Figure 3:**
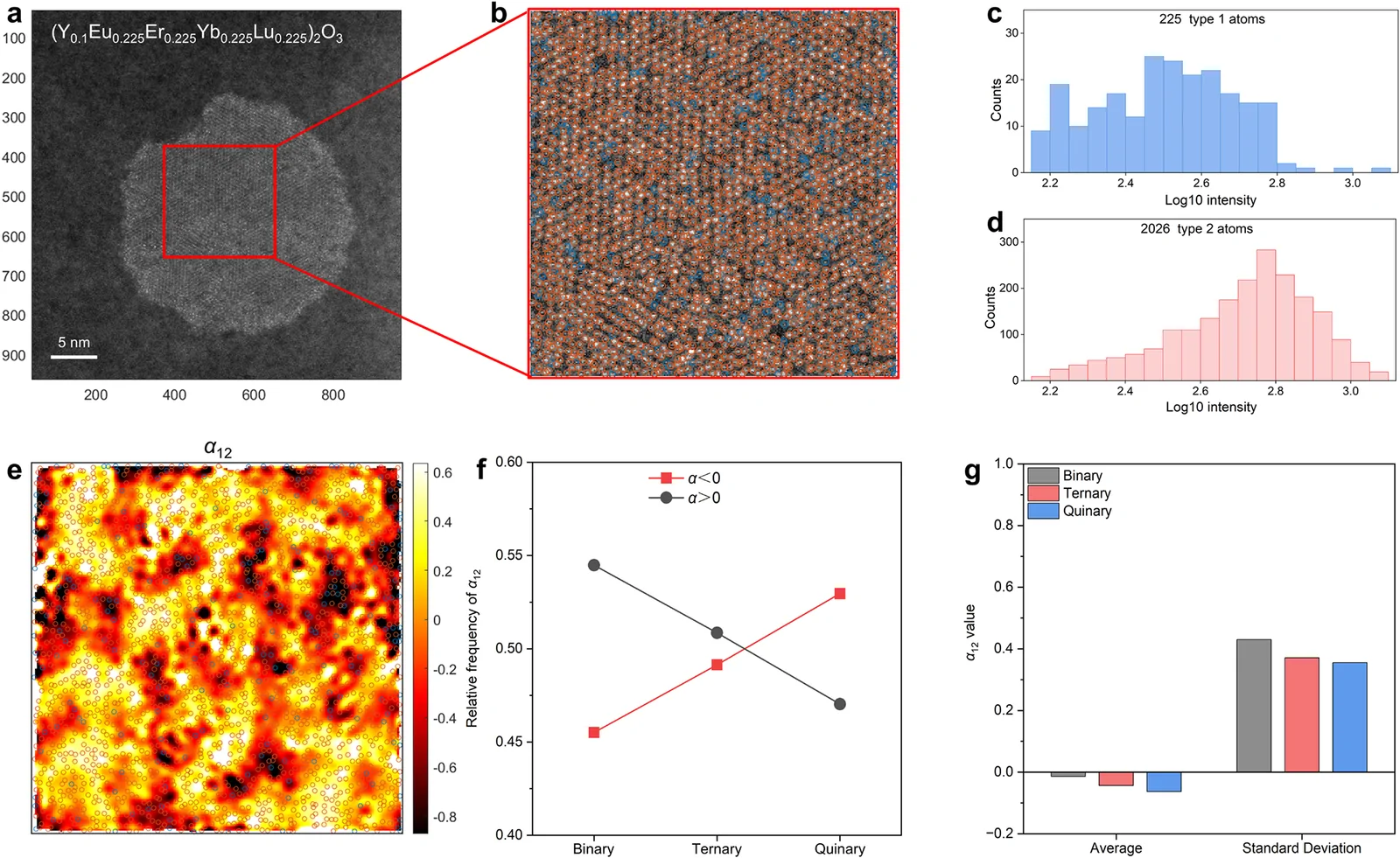
Local chemical ordering analysis of HE-REO nanoplatelets. (a) Aberration-corrected HAADF-STEM image of quinary (Y_0.1_Eu_0.225_Er_0.225_Yb_0.225_Lu_0.225_)_2_O_3_ HE-REO nanoplatelets. (b) Magnified atomic-resolution HAADF-STEM image and corresponding binary-type classification map based on atomic-column intensity contrast, in which type 1 and type 2 atomic columns are distinguished according to relative intensities. Statistical analysis of the identified (c) type 1 and (d) type 2 atomic columns. (e) Spatial maps, (f) relative frequency distributions and (g) average values and standard deviations of the Warren–Cowley CSRO parameter for heteroelement short-range ordering, α_12_, for binary, ternary and quinary HE-REO nanoplatelets.

The local chemical ordering was quantified using CSRO parameters calculated from nearest-neighbor atomic configurations.^39,40,52^ In this binary-type description, α_12_ describes heteroelement correlations between Y and heavier RE columns, whereas α_11_ and α_22_ describe Y–Y and RE–RE correlations, respectively. The positive α_11/_α_22_ values suggest a local segregation of type 1/2 atoms. The α_12_ denotes the Y-RE correlation where a negative value suggests an intermixing between the two species. The spatial maps of α_12_, α_11_ and α_22_ for the quinary nanoplatelets reveal a heterogeneous chemical-ordering landscape across the lattice (Figures 3e, S18a and S18b). The α_12_ map contains extended regions with negative values, indicating locally favored Y–RE intermixing. In contrast, α_11_ and α_22_ maps show localized fluctuations associated with Y–Y and RE–RE correlations. These variations remain confined to short length scales and do not develop into long-range ordered domains, indicating that the nanoplatelets retain chemically disordered local environments while exhibiting spatially fluctuating local chemical order.

Comparison among the binary, ternary and quinary compositions reveals a systematic evolution of CSRO with increasing configurational entropy. The most prominent trend is observed for α_12_, which shows a monotonic increase in the fraction of negative values, increasing from 0.455 (binary) to 0.491 (ternary) and 0.530 (quinary), accompanied by a corresponding decrease in the positive fraction (Figure 3f). Notably, the dominant population shifts from positive α_12_ values in the binary and ternary systems to negative α_12_ values in the quinary composition, indicating a transition from suppressed Y-RE intermixing to preferential Y-RE intermixing as the configurational entropy increases. Since negative α_12_ values correspond to favorable heteroelement mixing, this trend directly demonstrates a progressive enhancement of heteroelement short-range ordering with increasing configurational entropy. Consistently, the average α_12_ value shifts toward more negative values from −0.0139 (binary) to −0.0630 (quinary), confirming an increasing deviation from random mixing behavior (Figure 3g). In addition, the standard deviation decreases from 0.430 to 0.355, indicating a narrowing of the distribution and therefore a more homogeneous heterocoordination environment at higher component numbers (Figure 3g). Together, these results demonstrate that heteroelement short-range ordering not only becomes stronger but also progressively more spatially uniform as the system evolves toward higher-entropy compositions.

The α_11_ and α_22_ parameters provide complementary evidence for this ordering evolution. For α_11_, the fraction of random-like environments decreases markedly with increasing component number, while the fraction corresponding to suppressed Y–Y correlations increases more strongly than that associated with Y–Y clustering (Figure S18c). This suggests that Y atoms are increasingly incorporated into nonrandom local coordination environments dominated by neighboring RE species rather than forming Y-rich local regions. For α_22_, the average value decreases and the distribution narrows as the composition evolves from binary to quinary (Figure S18f), indicating a gradual homogenization of the RE-rich sublattice. Thus, the RE network does not appear to serve as the primary source of CSRO. Instead, it provides a more statistically balanced framework that accommodates the increasing preference for Y–RE heterocoordination.

Taken together, the binary-type CSRO analyses show that increasing configurational entropy does not simply enhance all types of local ordering simultaneously. Rather, the dominant ordering pathway is the progressive development of Y–RE heteroelement short-range ordering, accompanied by depletion of random Y-centered environments and homogenization of the RE-rich framework. Therefore, our analysis supports the development of Y-centered heteroelement short-range ordering as compositional complexity increases. These results demonstrate that colloidally synthesized, ultrathin HE-REO nanoplatelets provide a structurally defined platform for directly probing how configurational entropy regulates local atomic arrangements in HEOs. Note that conventional powder XRD mainly probes the long-range average crystal structure and is not sufficiently sensitive to directly resolve the Y-centered binary-type CSRO identified by HAADF-STEM analysis. Although CSRO may in principle contribute to weak diffuse scattering or peak broadening, these subtle effects are difficult to separate from the intrinsic broadening caused by the ultrathin nature of the nanoplatelets. Further work combining the demonstrated composition-design strategy with higher-chemical-sensitivity techniques will be needed to resolve element-specific short-range ordering of all lanthanide–lanthanide correlations.

### Formation Mechanism of HE-REO Nanoplatelets

Understanding how high-entropy materials nucleate and grow at the nanoscale is pivotal for controlling their structure, composition and local atomic arrangement. To elucidate the formation pathway of HE-REO nanoplatelets, aliquot samples of (YEuErYbLu)_2_O_3_ were collected at different reaction stages,^32^ and analyzed from both structural and compositional perspectives. Temperature-dependent synthesis studies reveal a continuous evolution from small nuclei (290 °C for 0 min) to petal-like intermediates (340 °C for 0 min), followed by irregular disk-like assemblies (340 °C for 15 min) and finally round, well-defined 2D nanoplatelets (340 °C for 60 min.) (Figure 4). This evolution suggests a kinetically controlled anisotropic growth process, in which initially formed nanoscale domains progressively coalesce into structurally coherent nanoplatelets. Such domain merging and shape refinement are consistent with a nonclassical growth pathway, rather than direct isotropic particle growth.

**Figure 4:**
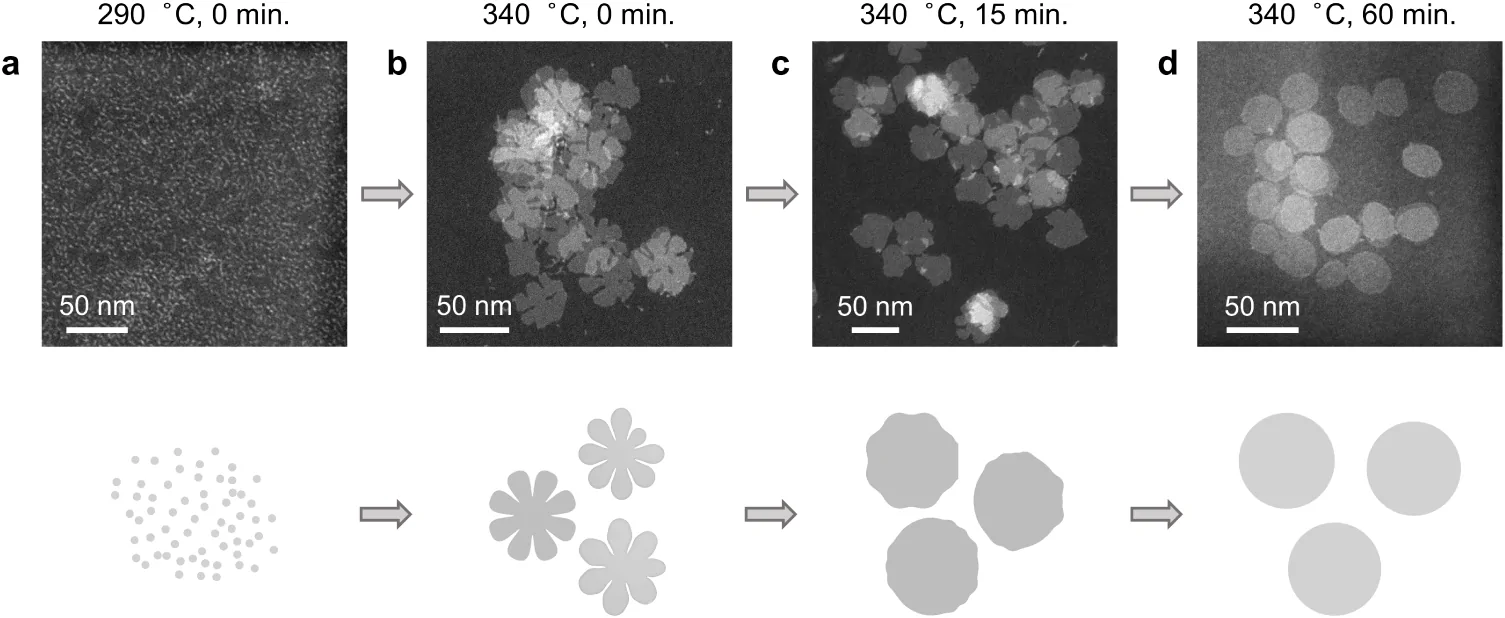
Formation pathway of HE-REO nanoplatelets. Temperature- and time-dependent aliquot studies showing the HAADF-STEM images of (YEuErYbLu)_2_O_3_ HE-REO nanoplatelets at the reaction stage of (a) 290 °C for 0 min, (b) 340 °C for 0 min, (c) 340 °C for 15 min, and (d) 340 °C for 60 min during the synthesis of the (YEuErYbLu)_2_O_3_ HE-REO nanoplatelets. Schematic illustration of the morphological evolution at each stage is also provided.

Elemental analysis of aliquot samples further reveals that Y, Eu, Er, Yb and Lu remain close to the nominal equimolar ratio throughout the entire growth stages, with each element maintained at approximately 20% (Figures S19–S21). This nearly invariant composition indicates that multication incorporation is established at the earliest nucleation stage and preserved during subsequent morphological evolution. Therefore, the formation of HE-REO nanoplatelets proceeds through simultaneous multication nucleation followed by anisotropic growth, rather than through sequential incorporation or postnucleation compositional equilibration. The growth pathway differs from previously reported colloidal HEA nanoparticles, where differences in reduction potentials often lead to sequential nucleation, element-specific enrichment/depletion or subsequent exchange processes during particle growth.^53,54^ It also contrasts with some colloidal HEO systems in which diffusion-assisted redistribution and surface-selective incorporation contribute to particle maturation and progressive evolution toward the final composition.^32^ For the HE-REO system in our case, the similar valence states, ionic sizes and oxide-formation tendencies of Y and rare-earth cations likely lead to comparable nucleation barriers, enabling concurrent incorporation of multiple cations into the nascent oxide lattice. Once incorporated, the limited long-range diffusion under colloidal growth conditions helps preserve the initial multication arrangement while the particle morphology continues to evolve. The distinct formation mechanism for HE-REO nanoplatelets also highlights the advantage of colloidal synthesis for constructing compositionally uniform HEO nanostructures, where homogeneous multication distributions can be established early while size, shape and crystallographic coherence are refined during growth.

### Surpassing Elemental Miscibility Limits through High-Entropy Design

Having established the homogeneous incorporation of chemically compatible rare-earth cations, we next used nonrare-earth cations to probe the balance between configurational entropy and mixing enthalpy in HE-REO nanoplatelets. Because chemically dissimilar cations impose larger enthalpic penalties through lattice distortion, charge imbalance and coordination mismatch, their incorporation provides a direct test of whether high-entropy design can stabilize multication oxide lattices beyond the rare-earth-only composition space.^21^ Sr^2+^ was first selected as a representative immiscible cation because of its distinct valence state and much larger ionic radius compared with trivalent rare-earth cations. A low-entropy ternary composition, (Y_0.33_Eu_0.33_Sr_0.33_)_2_O_3_, was synthesized as a control. As shown in Figure 5a (Figures S22 and S23a–b), the XRD pattern of this ternary sample contains additional diffraction features that can be assigned to the secondary SrEu_2_O_4_ and SrY_2_O_4_ phases. This result suggests that Sr^2+^ cannot be effectively incorporated into the REO host lattice under low-configurational-entropy conditions.

**Figure 5:**
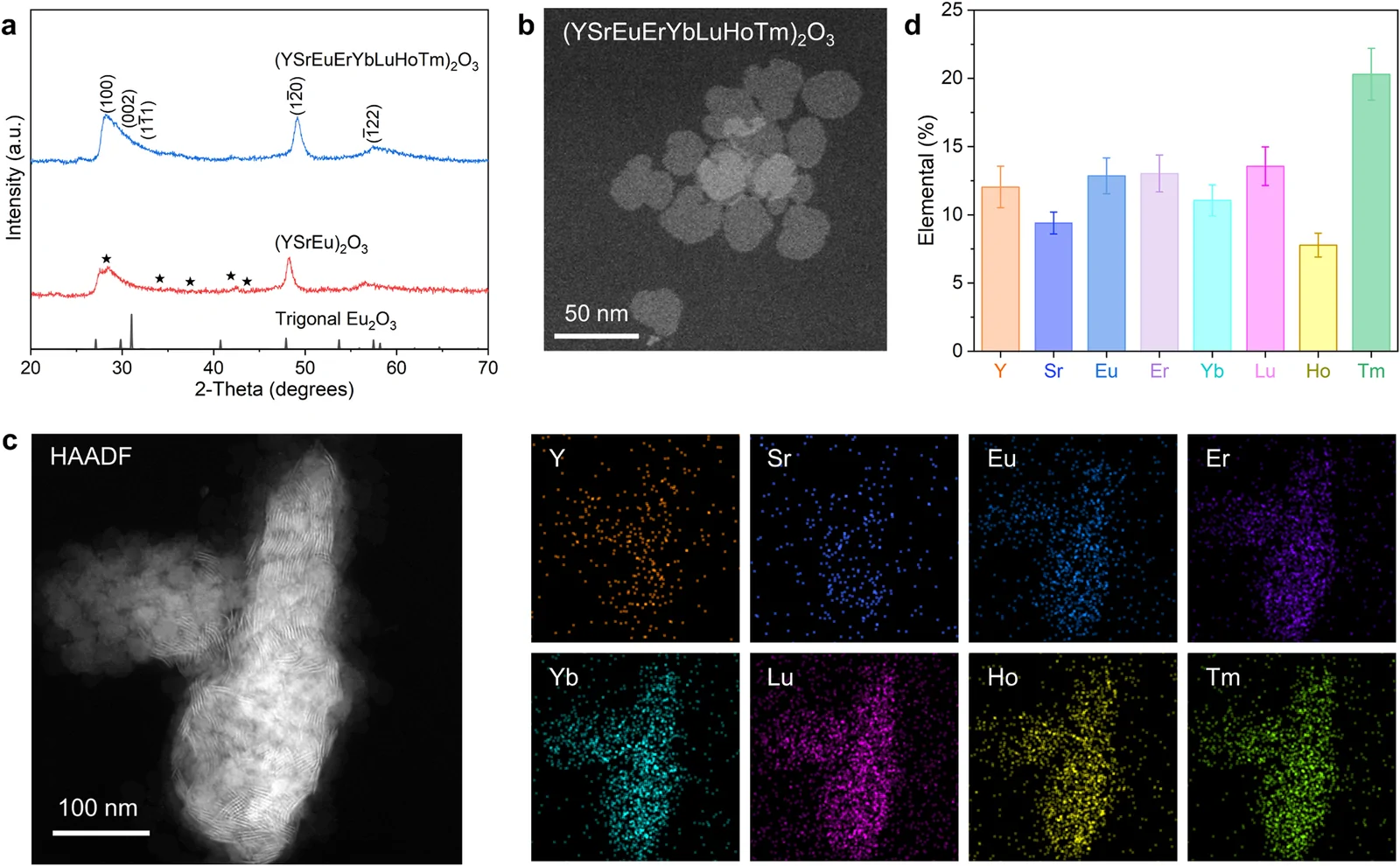
Entropy-assisted incorporation of Sr into HE-REO nanoplatelets. (a) XRD patterns of low-entropy ternary (YSrEu)_2_O_3_ and high-entropy octonary (YSrEuErYbLuHoTm)_2_O_3_ nanoplatelets, with the reference pattern for bulk trigonal Eu_2_O_3_ shown for comparison. (b) HAADF-STEM image, (c) STEM-EDS elemental maps, and (d) elemental compositions of (YSrEuErYbLuHoTm)_2_O_3_ nanoplatelets.

To increase the entropy contribution to surpass the miscibility hindrance, an octonary composition, (Y_0.125_Sr_0.125_Eu_0.125_Er_0.125_Yb_0.125_Lu_0.125_Ho_0.125_Tm_0.125_)_2_O_3_, was synthesized under otherwise identical conditions. In contrast to the ternary sample, the octonary product exhibits a single-phase diffraction pattern consistent with the trigonal REO lattice, with no detectable impurity peaks (Figure 5a). This comparison indicates that increasing configurational entropy facilitates Sr^2+^ incorporation into the REO lattice. To distinguish this entropy effect from a simple Sr-concentration effect, we further prepared a nonequimolar octonary composition with the same Sr content of 12.5 at.% but lower configurational entropy, (Eu_0.5_Sr_0.125_Y_0.0625_Ho_0.0625_Er_0.0625_Tm_0.0625_Yb_0.0625_Lu_0.0625_)_2_O_3_. Unlike the equimolar octonary sample, this lower-entropy composition shows additional diffraction peaks assignable to Sr-containing binary oxides, demonstrating that Sr segregation occurs when the configurational entropy is reduced while the Sr concentration remains unchanged (Figure S23c). These results confirm that Sr incorporation is governed by an entropy-assisted stabilization effect rather than simply by dilution of Sr concentration. TEM images show that the 2D nanoplatelet morphology is preserved upon Sr incorporation (Figure 5b). STEM-EDS elemental mapping further confirms that all constituent elements, including Sr, are homogeneously distributed throughout the nanoplatelet without evident segregation (Figure 5c). Quantitative EDS analysis shows that the overall composition is in reasonable agreement with the nominal stoichiometry (Figures 5d and S24).

To further evaluate the generality of this high-entropy design strategy, other nonrare-earth elements including Mg, Ca, Ti, and Ga were introduced to the trigonal REO lattice (Figure S25). These elements can also be incorporated while retaining the nanoplatelet morphology, further supporting the compositional flexibility of this colloidal platform (Figures S26 and S27). Notably, the edge structure and shape of some nonrare-earth-containing HE-REO nanoplatelets differ from those comprised of only rare-earth compositions. For example, the Ca-containing nanoplatelets show less regular circular outlines, whereas the Ga-containing sample exhibits an elongated disk morphology. These morphological variations suggest that nonrare-earth species alter the edge passivation or facet-selective growth of the nanoplatelets, which can be attributed to the distinct reactivities and stabilities of the corresponding metal oleate precursors under colloidal reaction conditions.

These results demonstrate that high-entropy design can expand the accessible composition space of the HE-REO nanoplatelets by facilitating the incorporation of chemically distinct nonrare-earth cations. In conventional low-entropy oxides, heterovalent or size-mismatched cations often introduce large enthalpic penalties associated with lattice distortion, charge imbalance and local coordination mismatch, which can drive phase separation or secondary-phase formation.^1^ Increasing the number of components elevates the configurational entropy contribution to the Gibbs free energy through the −TΔS term, reducing the thermodynamic driving force for segregation and favoring a multication solid-solution state.^1,55,56^ For colloidal nanostructures, this entropy contribution is further coupled with the structural tolerance of the trigonal REO structure and ligand-regulated crystallization, which together help accommodate otherwise incompatible cations under mild solution-phase conditions. Therefore, configurational entropy acts not only as a stabilizing factor for HE-REOs, but also as a practical design parameter for extending the miscibility window of oxide nanostructures.

## Conclusions

In summary, we have developed a colloidal synthetic platform for monodisperse HE-REO nanoplatelets with controlled composition and local atomic structure. This approach enables multiple rare-earth cations to be readily incorporated into a single-crystalline trigonal lattice without observable phase separation or obvious grain-boundary formation. Atomic-resolution HAADF-STEM combined with intensity-based classification suggests that, within the Y-marker-based binary representation, increasing configurational entropy promotes a heteroelement-dominated short-range ordered state, manifested by enhanced Y–RE nearest-neighbor correlations. Aliquot studies further uncover a simultaneous nucleation and growth mechanism, distinct from the sequential nucleation, exchange or compositional redistribution mechanism previously reported for high-entropy nanomaterials. In addition, high-entropy design enables incorporation of chemically dissimilar cations such as Sr^2+^ into higher-entropy compositions, demonstrating that configurational entropy can help offset mixing enthalpy penalties and expand the miscibility window of RE-based oxides.

Compared with thermally derived HE-REO nanopowders, this colloidal route directly provides solution-dispersible and structurally well-defined nanoscale building blocks, thereby reducing aggregation and morphological heterogeneity while enabling further ligand exchange and self-assembly. The resulting HE-REO nanoplatelets therefore serve not only as a synthetic advance in high-entropy ceramic nanomaterials, but also as a model system for probing enthalpy–entropy compensation and local chemical ordering. Their colloidal dispersibility and ligand-exchange capability open opportunities for solution-processed HEO films with extended functionalities, including catalytic interfaces, bioactive materials and rare-earth-based optical devices. More broadly, extending this strategy to other oxide families may enable high-entropy ceramics to be designed with the same level of nanoscale precision comparable to that achieved in colloidal semiconductor and metal nanocrystals.

## Supplementary Material



## Data Availability

The data supporting this study are available in the main text and the Supporting Information.

## Notes

Additional data, including X-ray emission lines, ionic radii, interplanar spacings, thickness measurements, XRD characterization, STEM-EDS elemental mapping, EDS spectra, ICP-OES analysis, CSRO calculations, and statistical analysis (PDF)
