# Supplementary material for: Colloidal Synthesis of Monodisperse High-Entropy Rare-Earth Oxide Nanoplatelets
Source: ACS Nanosci Au. 2026 Sep 4;6(5):1009–20. doi: 10.1021/acsnanoscienceau.6c00114 (PMC13693176; doi:10.1021/acsnanoscienceau.6c00114)
Supplement: Supplementary file 1 [file ng6c00114_si_001.pdf]

Supporting Information

Colloidal Synthesis of Monodisperse High-Entropy Rare-Earth Oxide Nanoplatelets

*Hanxin Fu[a], Lingchao Wang[b], Nachuan Li[a], Yi Zhou[a], Siyu Wang[b], Lianpeng Song[a], Yakun Yuan[b], Yifan Sun[a],**

[a]Frontiers Science Center for Transformative Molecules, State Key Laboratory of Synergistic Chem-Bio Synthesis, School of Chemistry and Chemical Engineering, and Zhangjiang Institute for Advanced Study, Shanghai Jiao Tong University, Shanghai 200240, China.

[b]Future Material Innovation Center, School of Materials Science and Engineering, Zhangjiang Institute for Advanced Study, and School of Physics and Astronomy, Shanghai Jiao Tong University, Shanghai 200240, China.

E-mail: sunyf@sjtu.edu.cn

Configurational entropy and average ionic radius calculation

The configurational entropy (S_{config}) of the multicomponent oxide systems was calculated according to[1]:

$$S_{\text{config}} = -R \sum_i x_i \ln x_i \qquad \text{Equation 1}$$

where R is the gas constant, and x_i is the atomic fraction of each cation species.

The average ionic radius (r_{avg}) was estimated using:

$$r_{\text{avg}} = \sum_i x_i \, r_i \qquad \text{Equation 2}$$

where r_i represents the ionic radius of each cation species and x_i is the corresponding atomic fraction.

The interplanar spacing corresponding to the $(1\bar{2}0)$ reflection from the XRD patterns using Bragg's law:

$$2d\sin\theta=\lambda \qquad \text{Equation 3}$$

where λ=1.54Å.

Supplementary Tables

Table S1. Principal L-series characteristic X-ray emission lines employed for EDS quantification of rare-earth elements.

Element	Lα1 (keV)	Lβ1 (keV)	Lβ2 (keV)	Lα2 (keV)	Lβ3 (keV)	Line for quantification
Eu	5.846	6.4565	6.8437	5.8163	6.5714	L lines
Er	6.9486	7.811	8.1903	6.9046	7.9395	L lines
Yb	7.4158	8.4019	8.7587	7.3673	8.5366	L lines
Lu	7.6556	8.7092	9.0491	7.6047	8.8468	L lines
Dy	6.4952	7.2481	7.6359	6.4576	7.3702	L lines
Tm	7.1803	8.1023	8.4684	7.1334	8.2312	L lines
Ho	6.7197	7.5263	7.9101	6.6796	7.653	L lines
Gd	6.0576	6.7131	7.1023	6.0256	6.8316	L lines
Tb	6.2728	6.9766	7.367	6.239	7.0967	L lines
Ce	4.8401	5.2629	5.6134	4.8221	5.3634	L lines

Table S2. Principal K-series characteristic X-ray emission lines used for EDS quantification of Y and non-rare-earth elements.

Element	Kα1 (keV)	Kα2 (keV)	Kβ1 (keV)	Line for quantification
Y	14.9584	14.8829	16.7381	K lines
Sr	14.165	14.0978	15.8355	K lines
Mg	1.2536			K lines
Ca	3.6917	3.6881	4.0127	K lines
Ti	4.5109	4.5049	4.9318	K lines
Ga	9.2517	9.2248	10.2642	K lines
Cu	8.0478	8.0279	8.9053	K lines
O	0.5249			K lines
C	0.2774			K lines
N	0.3924			K lines

Table S3. Ionic radii and valence states of cations incorporated in the HE-REO nanoplatelets. Ionic radii are based on the Shannon radii for six-fold coordination.

Cations	Valence state	Ionic radius (Å)
Eu	+3	0.947
Gd	+3	0.938
Tb	+3	0.923
Dy	+3	0.912
Ho	+3	0.901
Y	+3	0.900
Er	+3	0.890
Tm	+3	0.880
Yb	+3	0.868
Lu	+3	0.861
Sr	+2	1.180
Ca	+2	1.000
Mg	+2	0.720
Ga	+3	0.620
Ti	+4	0.605

Table S4. Average ionic radii and configurational entropy values of ternary, quinary, octonary, and denary REO systems.

Composition	Number of components	Configurational entropy	Average ionic radius (Å)
Eu_2O_3	1	0	0.947
$(YEuYb)_2O_3$	3	0.95R	0.907
$(YEuYbErLu)_2O_3$	5	1.57R	0.892
$(YEuYbErLuDyHoTm)_2O_3$	8	2.06R	0.895
$(YEuYbErLuDyHoTmGdTb)_2O_3$	10	2.30R	0.902

Table S5. Comparison of the $(1\bar{2}0)$ interplanar spacings obtained from iFFT analysis and XRD patterns for Eu_2O_3 and high-entropy REO nanoplatelets.

Sample	d (Å)-iFFT	d (Å)-XRD	Difference (Å)
Eu_2O_3	1.949	1.901	0.048
$(Y_{0.1}Eu_{0.9})_2O_3$	2.083	1.892	0.191
$(Y_{0.1}Eu_{0.45}Yb_{0.45})_2O_3$	1.967	1.866	0.101
$(Y_{0.1}Eu_{0.225}Yb_{0.225}Er_{0.225}Lu_{0.225})_2O_3$	1.920	1.852	0.068
$(YEuYbErLu)_2O_3$	1.984	1.845	0.139
$(YEuYbErLuDyHoTmGdTb)_2O_3$	1.899	1.864	0.035

The interplanar spacing corresponding to the $(1\bar{2}0)$ reflection from the XRD patterns using Bragg's law (Equation S3). Meanwhile, the lattice spacings were further measured from inverse FFT (iFFT) images generated from the $(1\bar{2}0)$ and $(\bar{1}20)$ reflections of atomic-resolution STEM images. The corresponding results are also summarized in Table S5. The lattice spacings obtained from iFFT and XRD show consistent compositional evolution, indicating good agreement between local lattice measurements from individual nanoplatelets and ensemble-averaged structural information from XRD.

Table S6. Statistical thickness analysis of Eu_2O_3 and representative HE-REO nanoplatelets.

Sample	Thickness (nm)	Number of Particles
Eu_2O_3	2.2 ± 0.4	100
$(Y_{0.1}Eu_{0.9})_2O_3$	2.2 ± 0.4	80
$(Y_{0.1}Eu_{0.45}Yb_{0.45})_2O_3$	1.9 ± 0.3	80
$(Y_{0.1}Eu_{0.225}Er_{0.225}Yb_{0.225}Lu_{0.225})_2O_3$	2.0 ± 0.3	80
$(YEuYb)_2O_3$	2.2 ± 0.4	80
$(YEuYbErLu)_2O_3$	1.5 ± 0.4	80
$(YEuYbErLuDyHoTm)_2O_3$	1.9 ± 0.4	80
$(YEuYbErLuDyHoTmGdTb)_2O_3$	1.5 ± 0.3	80

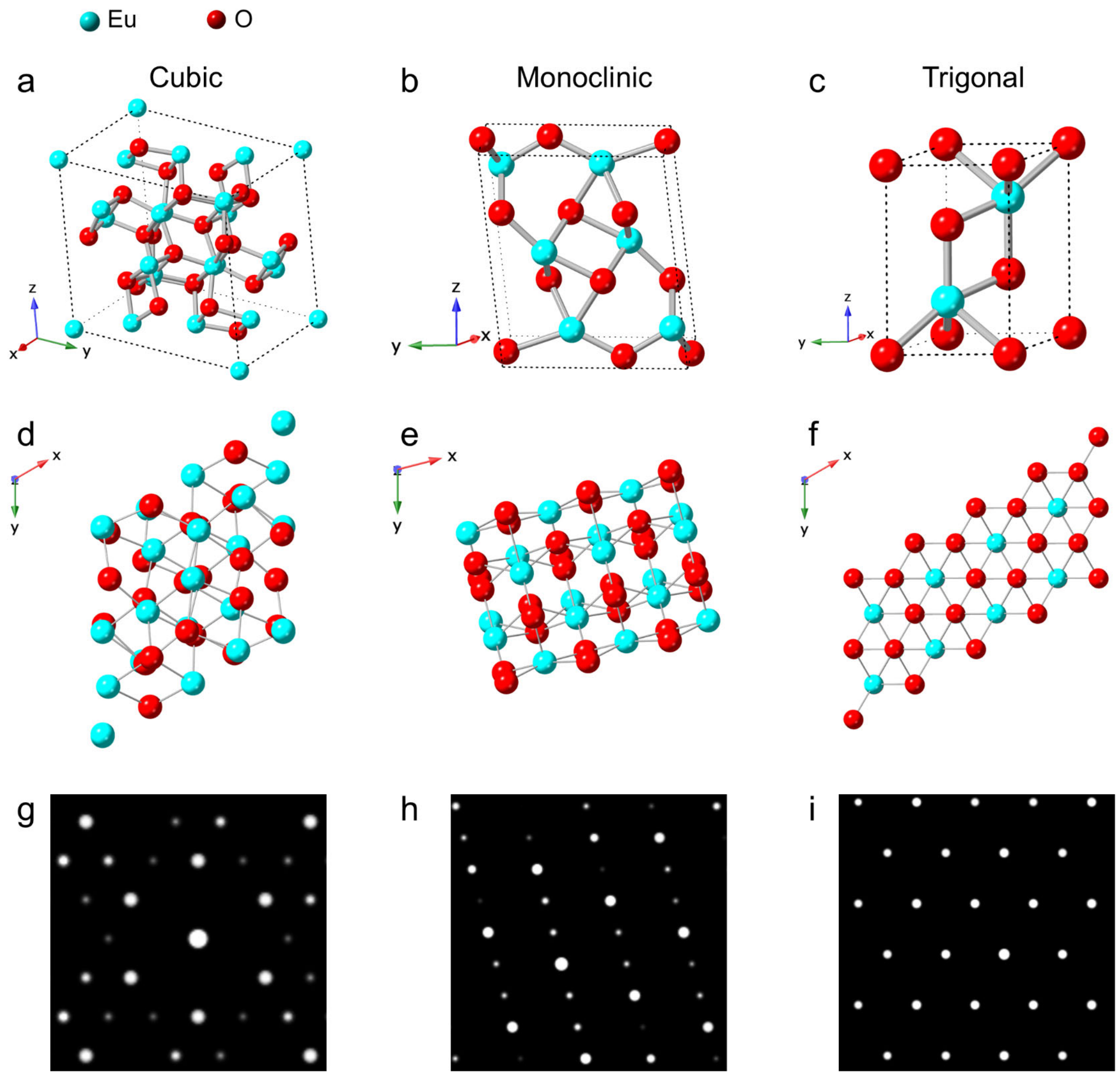

Figure S1. Unit-cell structural models of bulk Eu_2O_3 polymorphs: (a) cubic, (b) monoclinic and (c) trigonal Eu_2O_3. Crystal structural models viewed along the c-axis and their corresponding simulated FFT patterns for (d,g) cubic, (e,h) monoclinic and (f,i) trigonal Eu_2O_3.

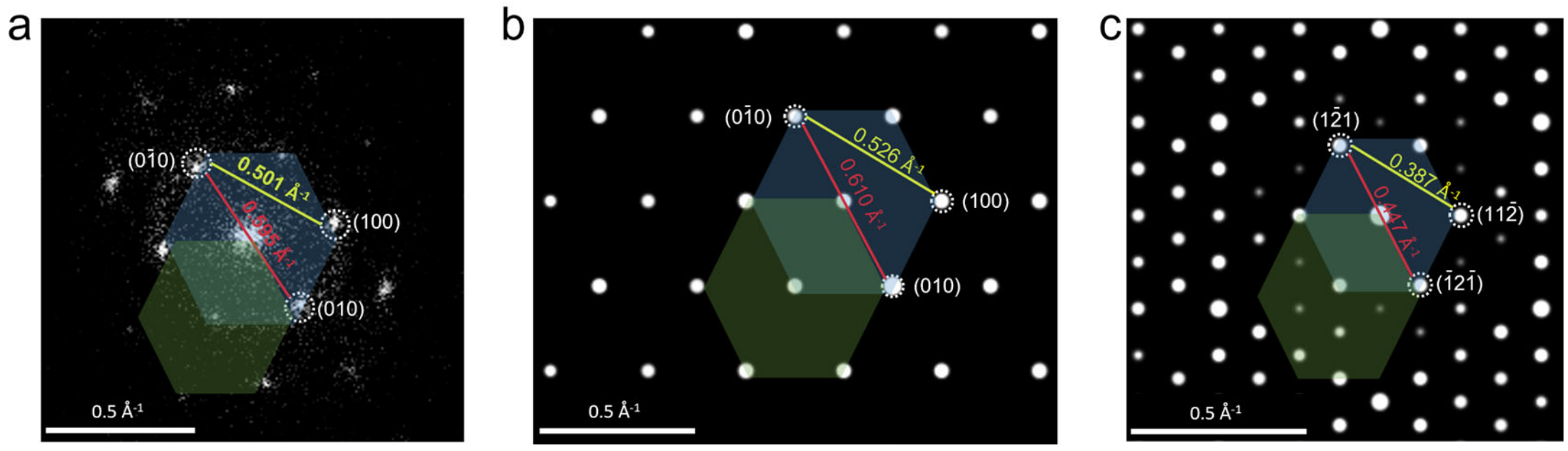

Figure S2. Comparison of experimental and simulated FFT patterns for phase identification of Eu_2O_3 nanoplatelets. (a) Experimental FFT pattern obtained from the atomic-resolution STEM image of the Eu_2O_3 nanoplatelet. (b) Simulated FFT pattern of the metastable trigonal Eu_2O_3 phase viewed along the [001] (c-axis) zone axis. (c) Simulated FFT pattern of cubic Eu_2O_3 viewed along the [111] zone axis.

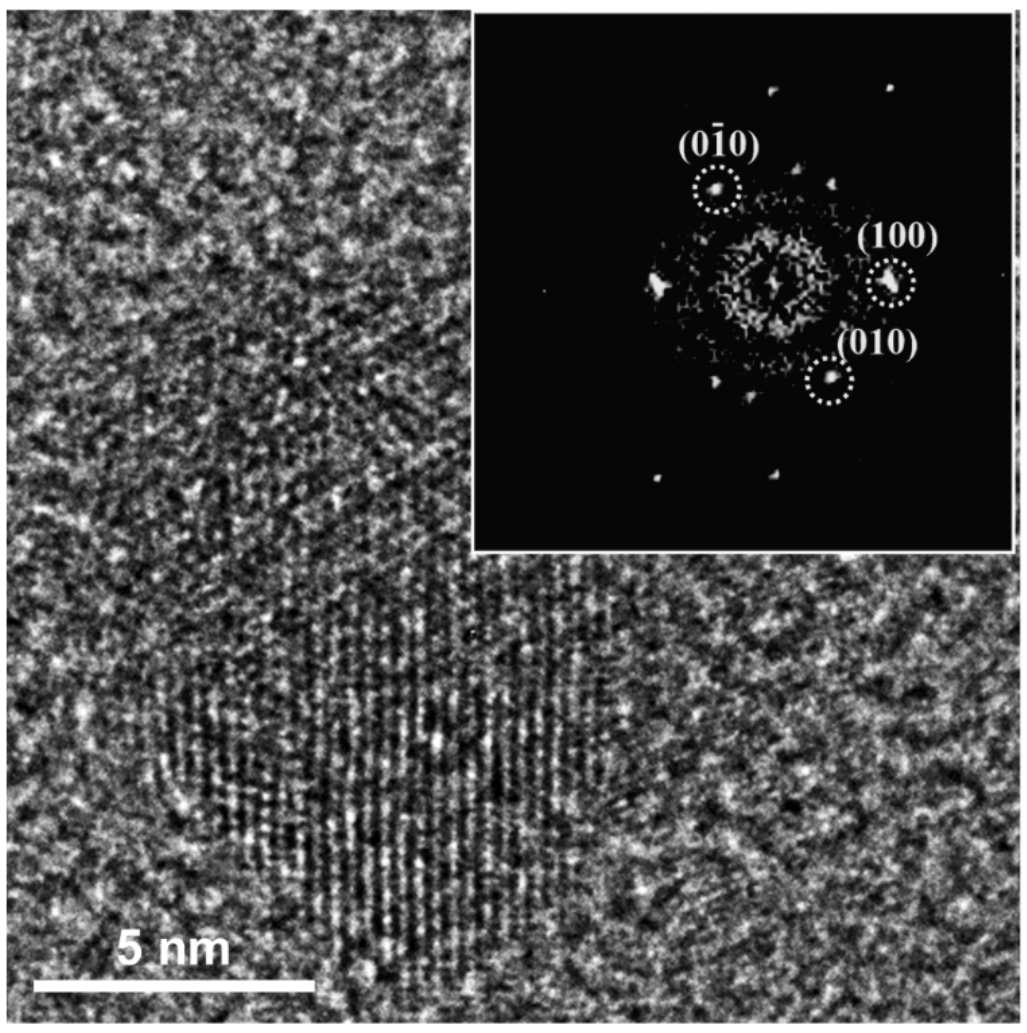

Figure S3. TEM image of trigonal Eu_2O_3 nanoplatelets upon annealing at 400 °C in air with the corresponding FFT pattern shown in the inset. The FFT retains the characteristic hexagonal symmetry after annealing, while the additional weak diffraction spots may arise from nanoplatelet stacking during thermal treatment.

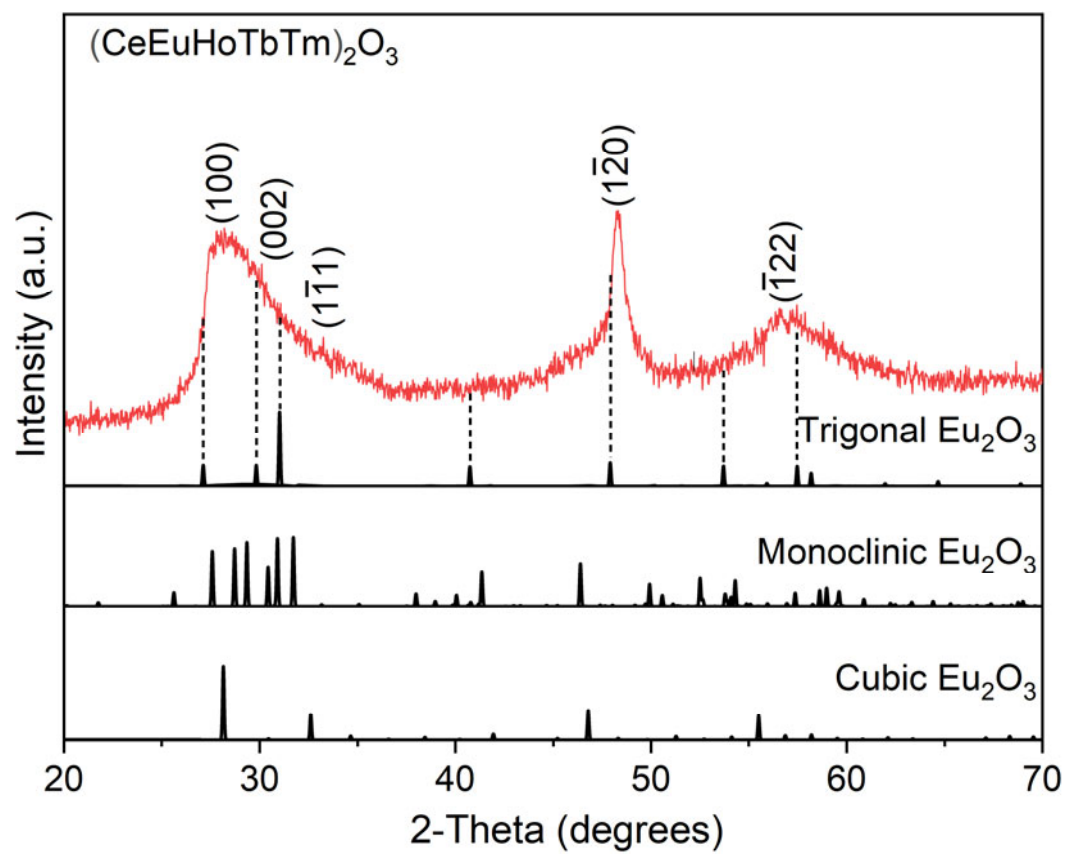

Figure S4. XRD pattern of colloidal (CeEuHoTbTm)$_2$O$_3$ nanoplatelets, with simulated patterns of bulk trigonal, monoclinic and cubic Eu_2O_3 for comparison.

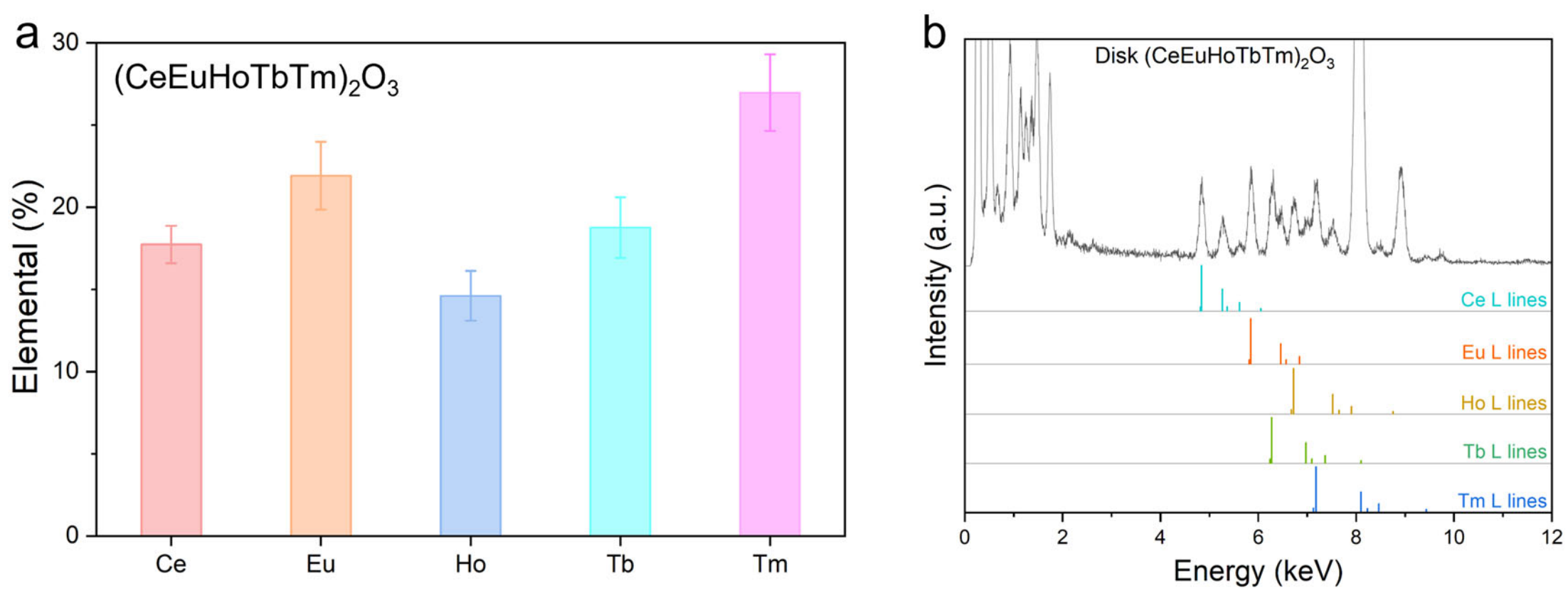

Figure S5. (a) Elemental composition. (b) STEM-EDS spectrum of colloidal (CeEuHoTbTm)$_2$O$_3$ nanoplatelets determined by STEM-EDS analysis.

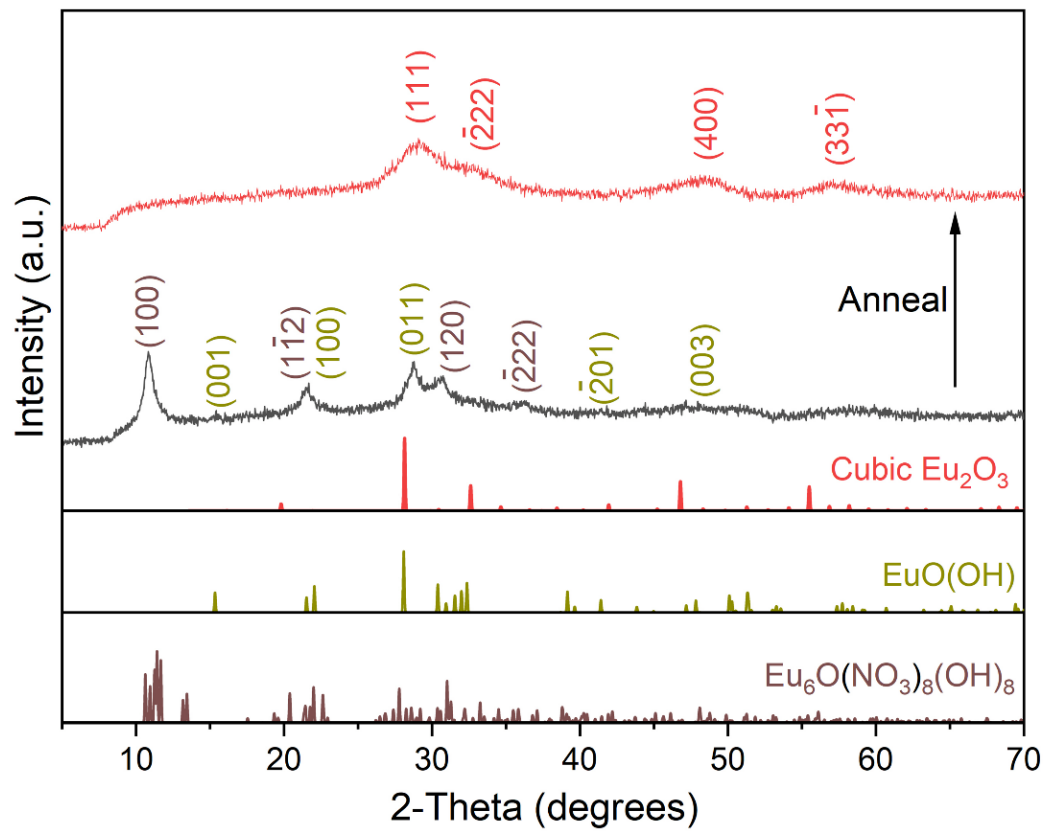

Figure S6. XRD pattern of the mixed-phase oxide product synthesized using the colloidal CeO_2 nanoparticle protocol.

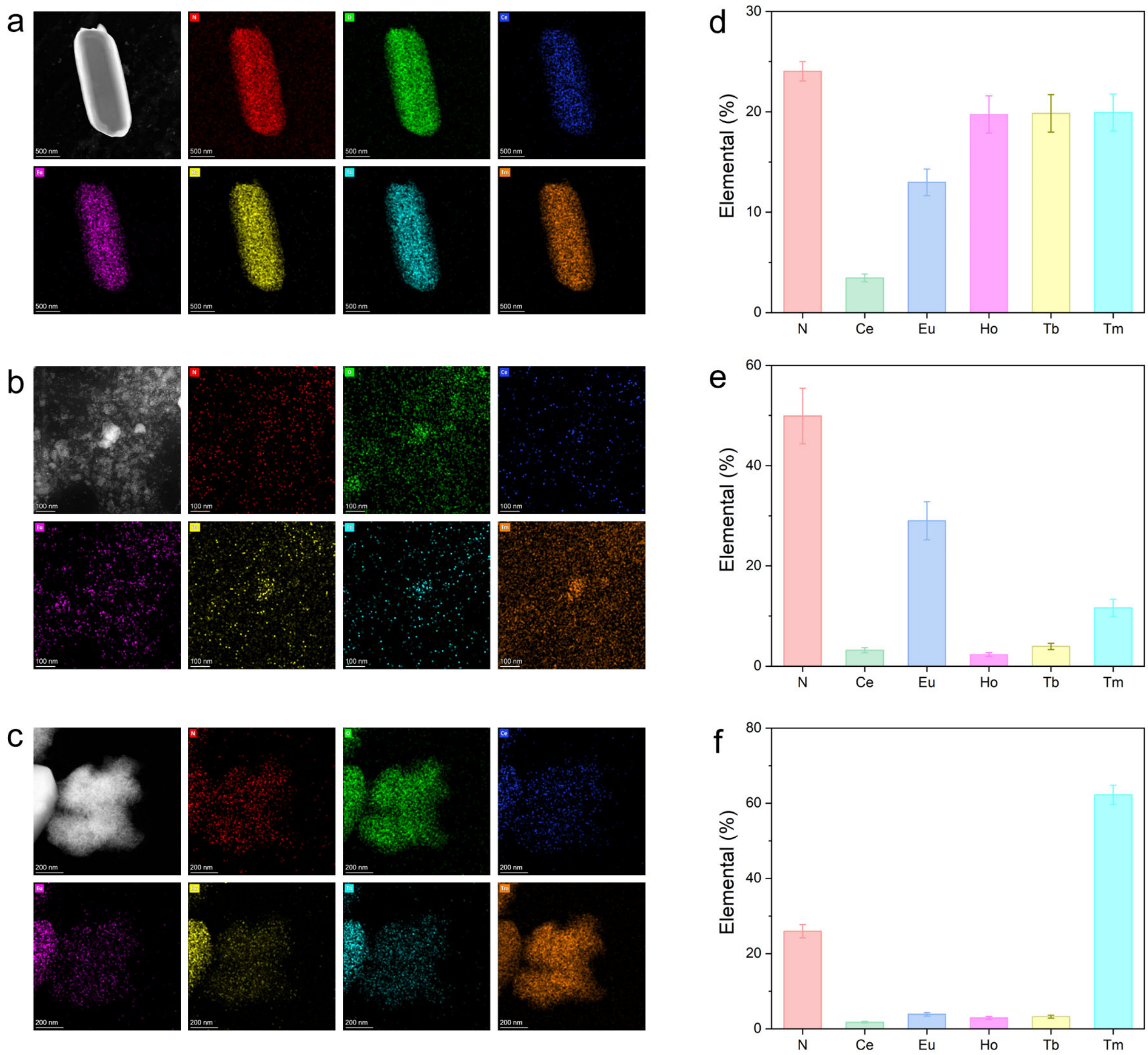

Figure S7. HAADF-STEM images, corresponding STEM-EDS elemental maps and calculated elemental compositions of the mixed-phase oxide product synthesized using the colloidal CeO_2 nanoparticle protocol, showing distinct domains of (a,d) nanorods approximately 2–3 μm in length, (b,e) irregular nanoplatelets containing precursor-derived species and (c,f) nanoscale clusters. The co-existence of distinct morphologies and observable elemental heterogeneity is consistent with the XRD results, suggesting that the stable cubic-phase route is unfavorable for directly producing single-phase HE-REO nanostructures under the colloidal synthesis conditions.

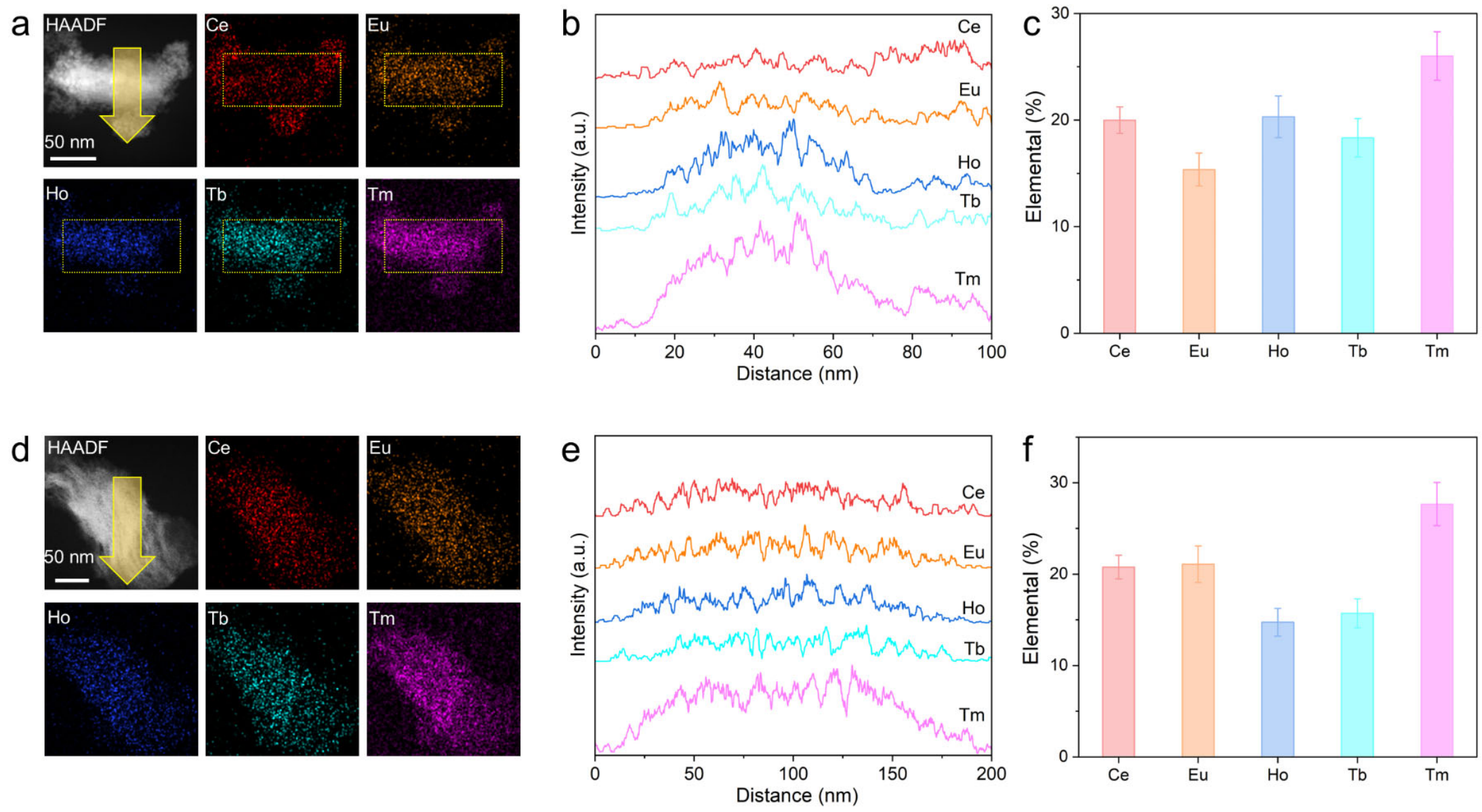

Figure S8. HAADF-STEM images, corresponding STEM-EDS elemental maps, line-scan profiles and elemental compositions of the annealed (300 °C, 5h) $(CeEuHoTbTm)_2O_3$ samples synthesized using the (a–c) cubic CeO_2 nanoparticle approach and (d–f) trigonal Eu_2O_3 nanoplatelet protocol, respectively. The line-scan profiles reveal pronounced elemental distribution heterogeneity in the sample obtained from the CeO_2 nanoparticle approach, whereas the sample synthesized using the Eu_2O_3 nanoplatelet protocol exhibits a uniform distribution of all constituent elements.

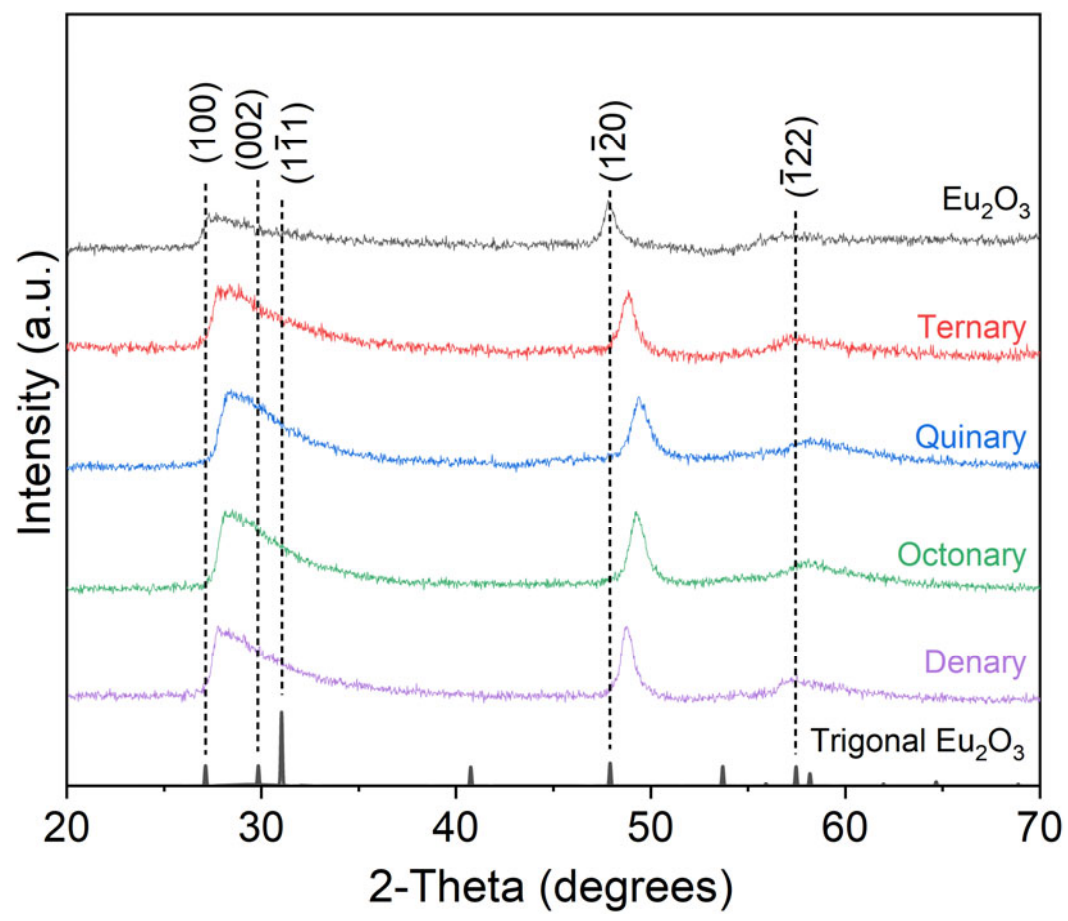

Figure S9. XRD patterns of colloidal Eu_2O_3, ternary, quinary, octonary, and denary HE-REO nanoplatelets.

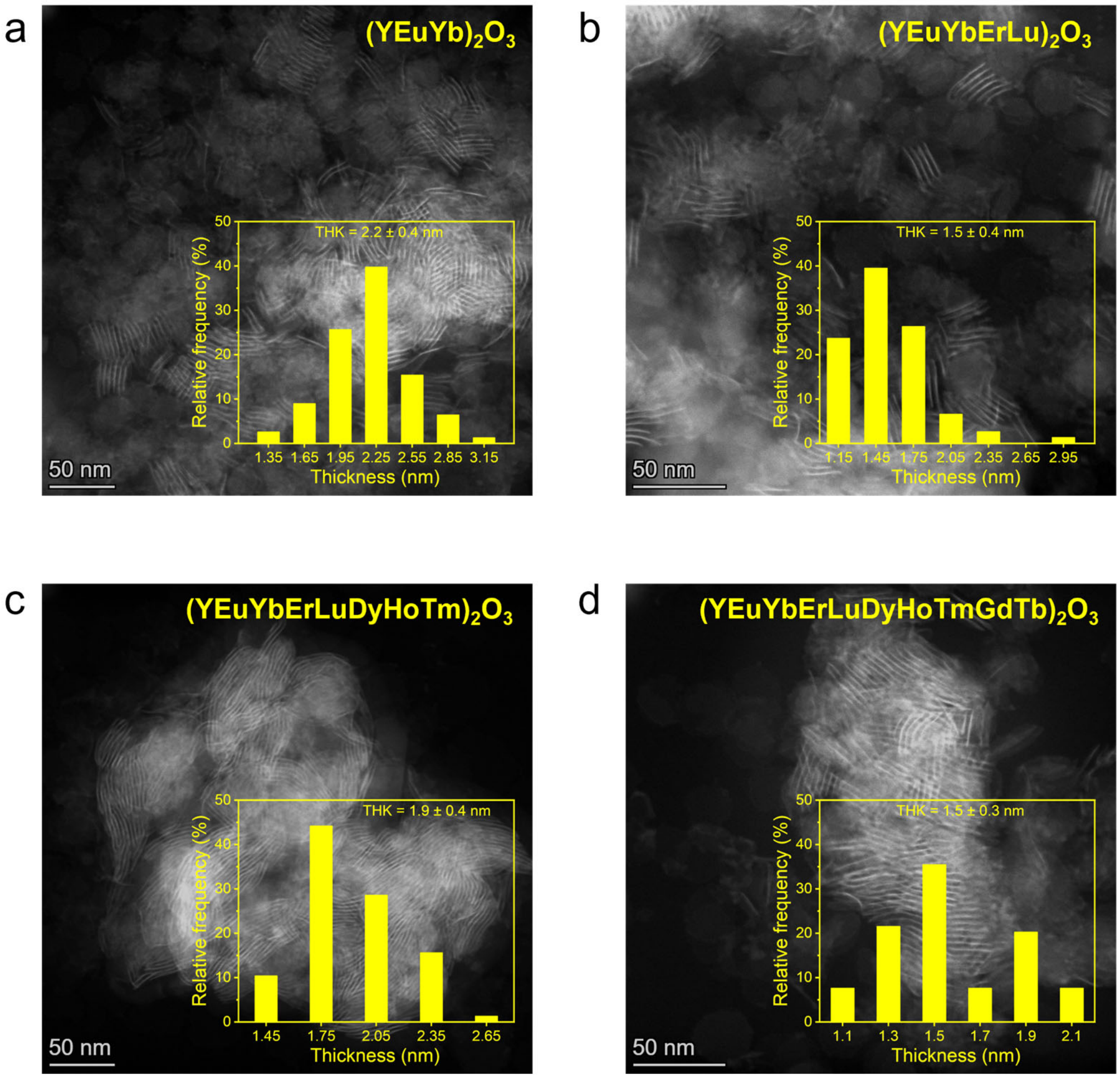

Figure S10. Side-view HAADF-STEM images with corresponding thickness distribution histograms of (a) ternary $(YEuYb)_2O_3$, (b) quinary $(YEuYbErLu)_2O_3$, (c) octonary $(YEuYbErLuDyHoTm)_2O_3$ and (d) denary $(YEuYbErLuDyHoTmGdTb)_2O_3$ nanoplatelets.

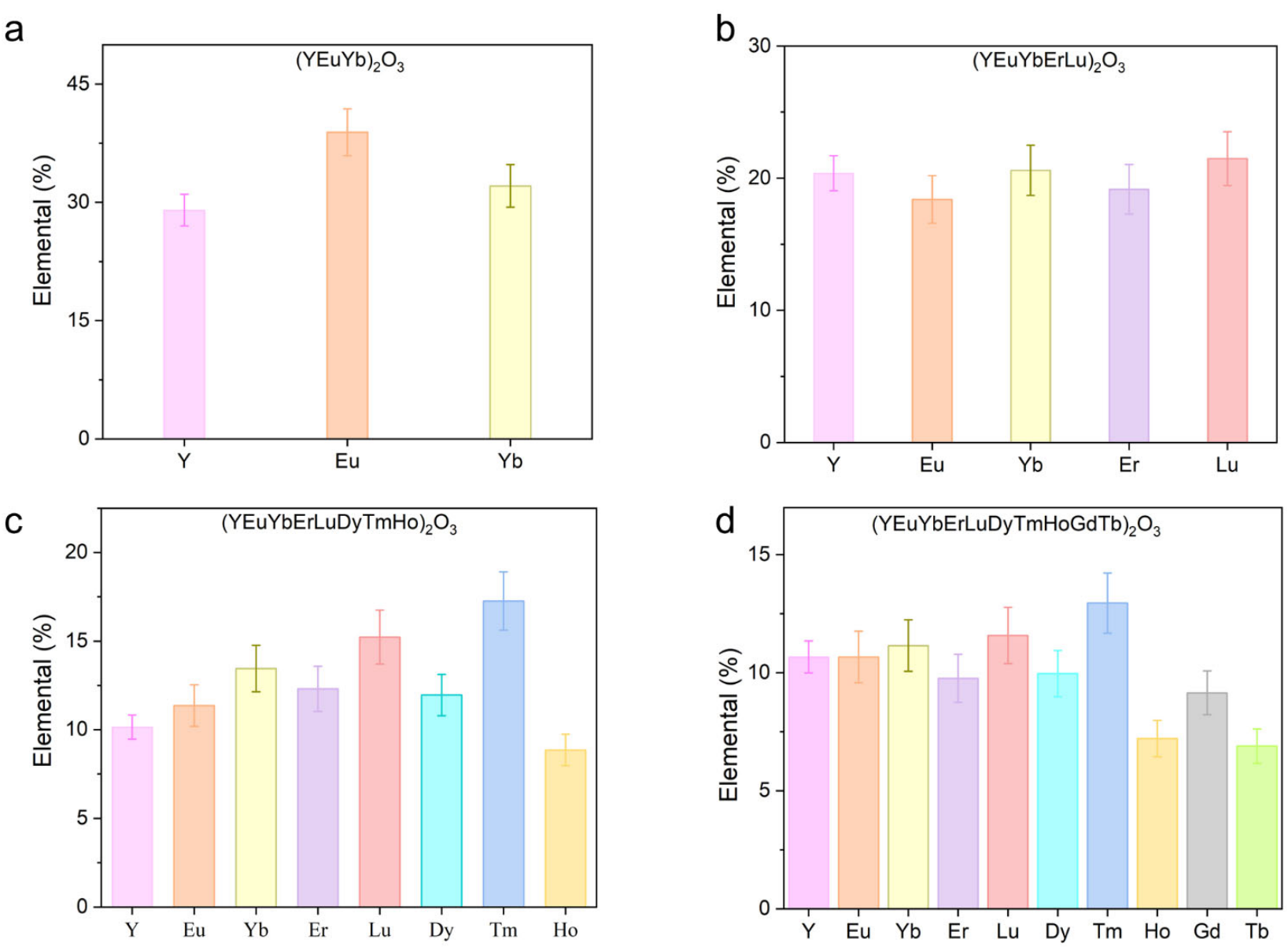

Figure S11. Elemental composition of (a) ternary, (b) quinary, (c) octonary and (d) denary HE-REO nanoplatelets determined using STEM-EDS.

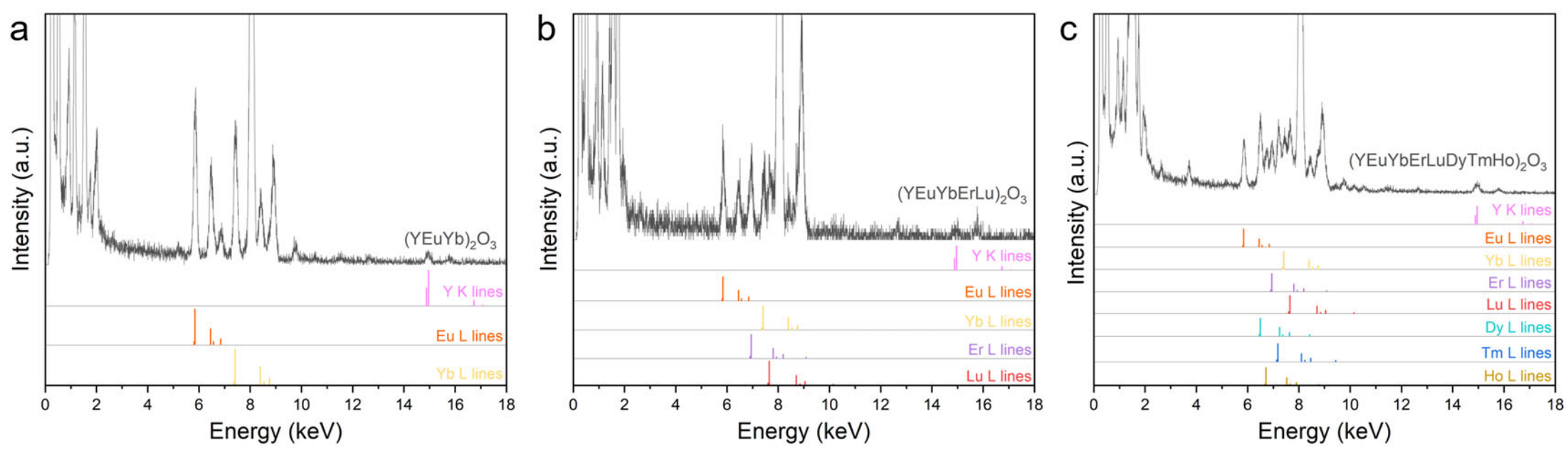

Figure S12. STEM-EDS spectra of (a) ternary $(YEuYb)_2O_3$, (b) quinary $(YEuYbErLu)_2O_3$, and (c) octonary $(YEuYbErLuDyHoTm)_2O_3$ nanoplatelets, with characteristic X-ray emission lines labeled for elemental identification.

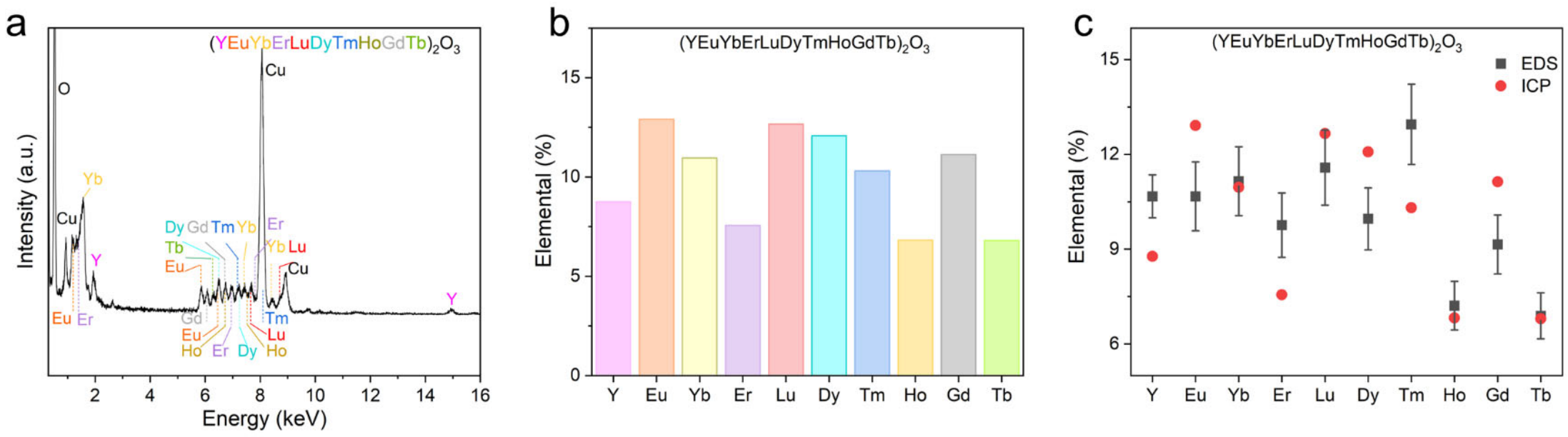

Figure S13. (a) STEM-EDS spectra, (b) elemental composition determined by ICP-OES, and (c) comparison of elemental compositions obtained from STEM-EDS and ICP-OES for denary HE-REO nanoplatelets. The overall compositional trends determined by STEM-EDS are generally consistent with those measured by ICP-OES. Minor discrepancies between the two methods mainly arise from their different sampling volumes and analytical principles. STEM-EDS analyzes selected local regions of individual nanoplatelets or nanoplatelet assemblies, whereas ICP-OES provides the bulk-averaged elemental composition of the fully dissolved sample. In addition, EDS quantification in multicomponent rare-earth systems is intrinsically challenging.[2] Rare-earth L-series emission lines are crowded within the 5–8 keV range, leading to partial peak overlap among neighboring elements, such as Eu Lα/Gd Lα and Gd Lβ/Ho Lα. Similar overlap effects may also affect Tb/Dy/Er and Tm/Yb/Lu quantification. In addition, Y was quantified using its high-energy K-series emission line, which may introduce additional statistical uncertainty due to reduced detection efficiency in Si-based EDS detectors, especially at low concentrations.

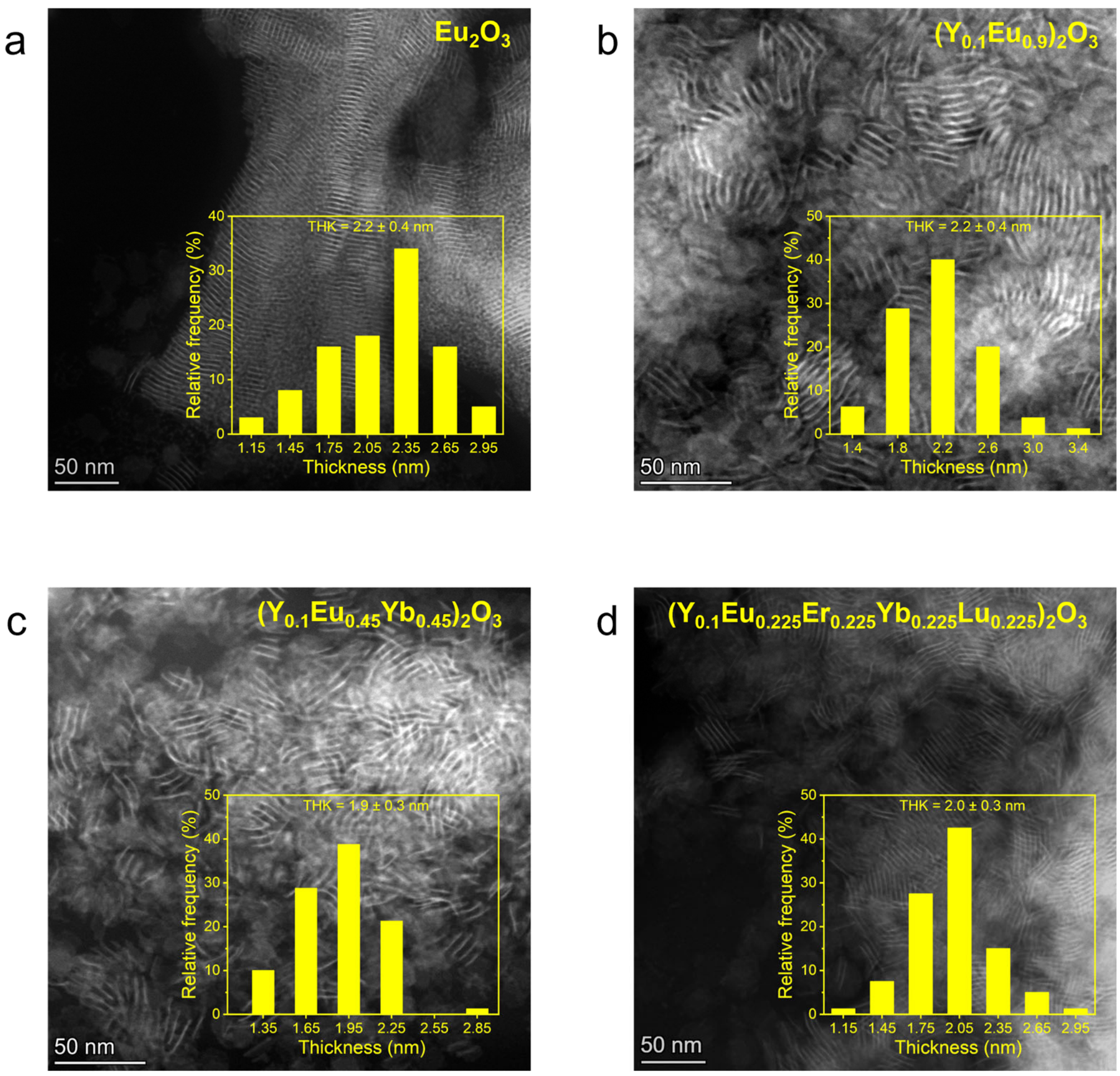

Figure S14. Side-view HAADF-STEM images with corresponding thickness distribution histograms of (a) Eu_2O_3, (b) $(Y_{0.1}Eu_{0.9})_2O_3$, (c) $(Y_{0.1}Eu_{0.45}Yb_{0.45})_2O_3$, and (d) $(Y_{0.1}Eu_{0.225}Er_{0.225}Yb_{0.225}Lu_{0.225})_2O_3$ nanoplatelets.

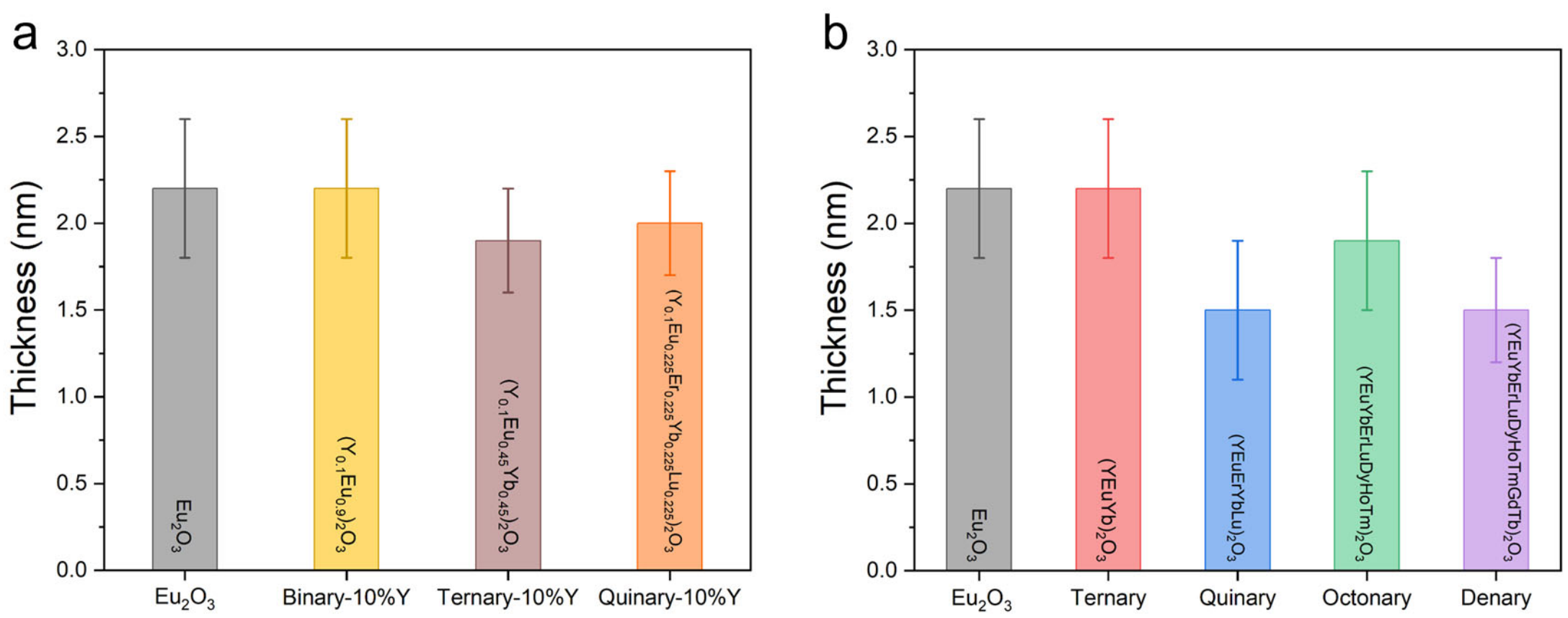

Figure S15. Comparison of the average thicknesses of nanoplatelets with different compositions.

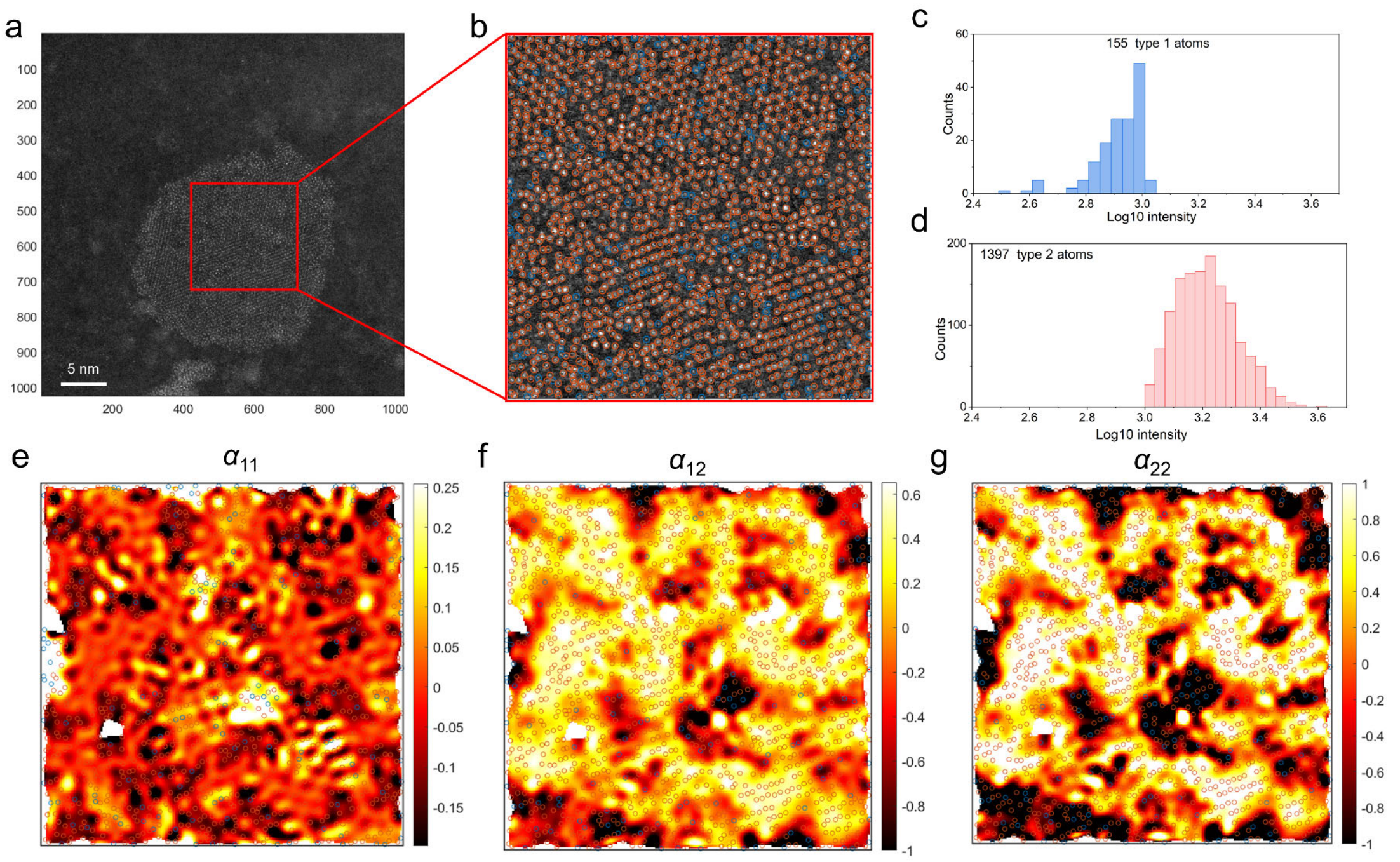

Figure S16. (a) Aberration-corrected HAADF-STEM image of binary $(Y_{0.1}Eu_{0.9})_2O_3$ nanoplatelets. (b) Magnified atomic-resolution HAADF-STEM image and corresponding binary-type classification map based on atomic-column intensity contrast, in which type 1 and type 2 atomic columns are distinguished according to relative intensities. Statistical analysis of the identified (c) type 1 and (d) type 2 atomic columns. (e) Spatial maps for the CSRO parameters: (e) α_{11}, (f) α_{12} and (g) α_{22}.

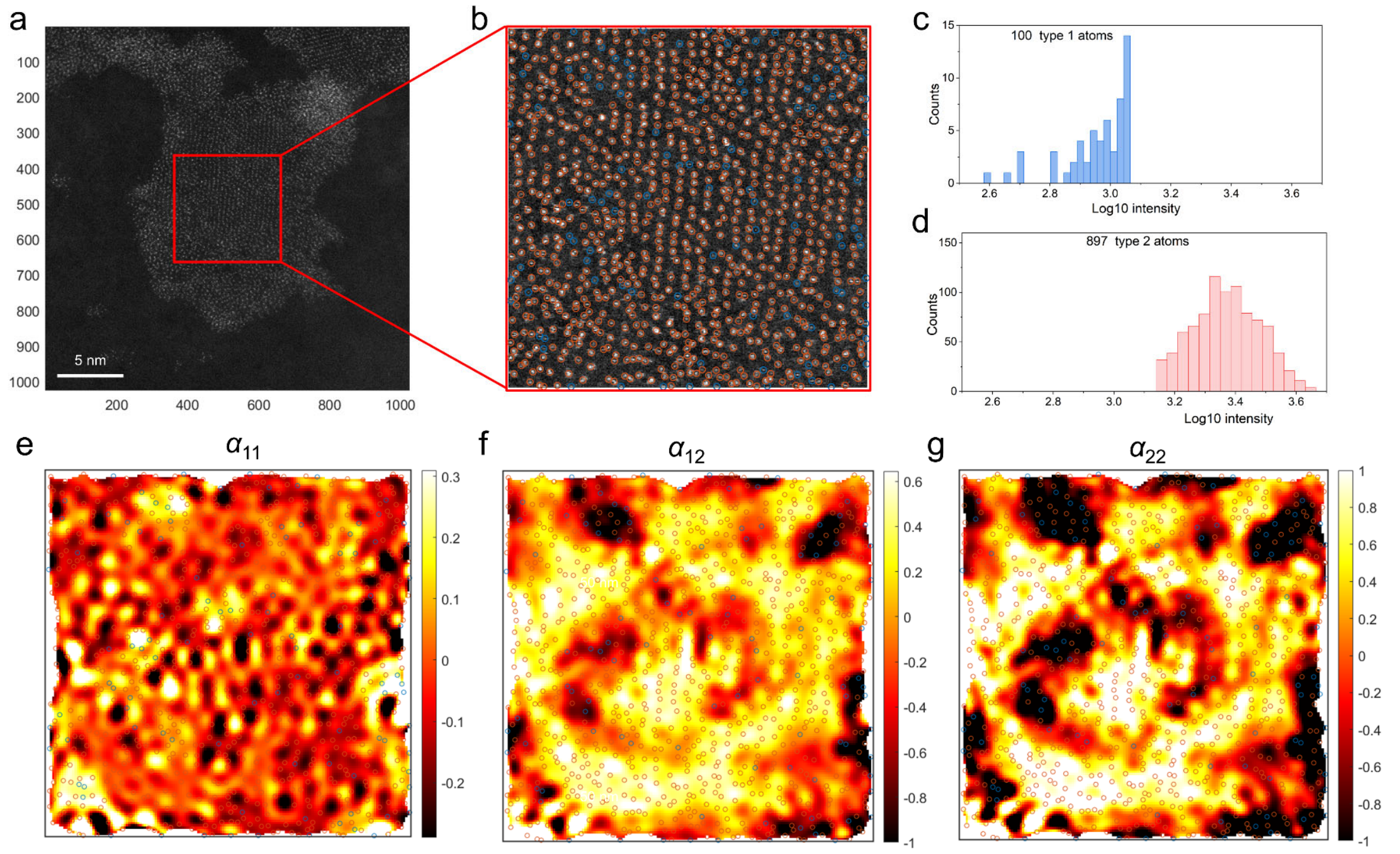

Figure S17. (a) Aberration-corrected HAADF-STEM image of ternary $(Y_{0.1}Eu_{0.45}Yb_{0.45})_2O_3$ nanoplatelets. (b) Magnified atomic-resolution HAADF-STEM image and corresponding binary-type classification map based on atomic-column intensity contrast. Statistical analysis of the identified (c) type 1 and (d) type 2 atomic columns. (e) Spatial maps for the CSRO parameters: (e) α_{11}, (f) α_{12} and (g) α_{22}.

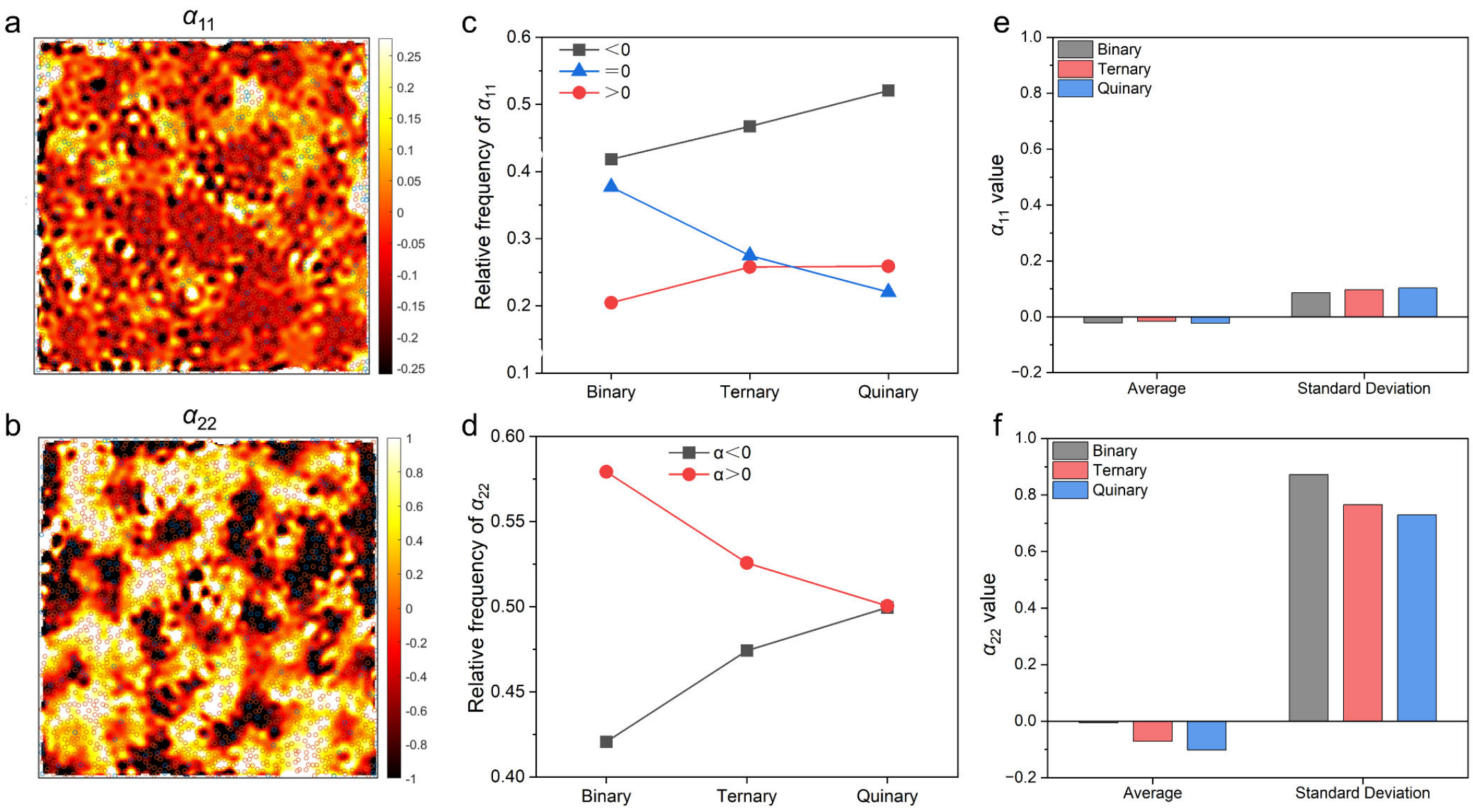

Figure S18. Spatial maps of the CSRO parameter (a) α_{11} and (b) α_{22} for quinary $(Y_{0.1}Eu_{0.225}Er_{0.225}Yb_{0.225}Lu_{0.225})_2O_3$ HE-REO nanoplatelets. The relative frequencies and average values with standard deviation of (c,e) α_{11} and (d,f) α_{22} for binary, ternary and quinary samples.

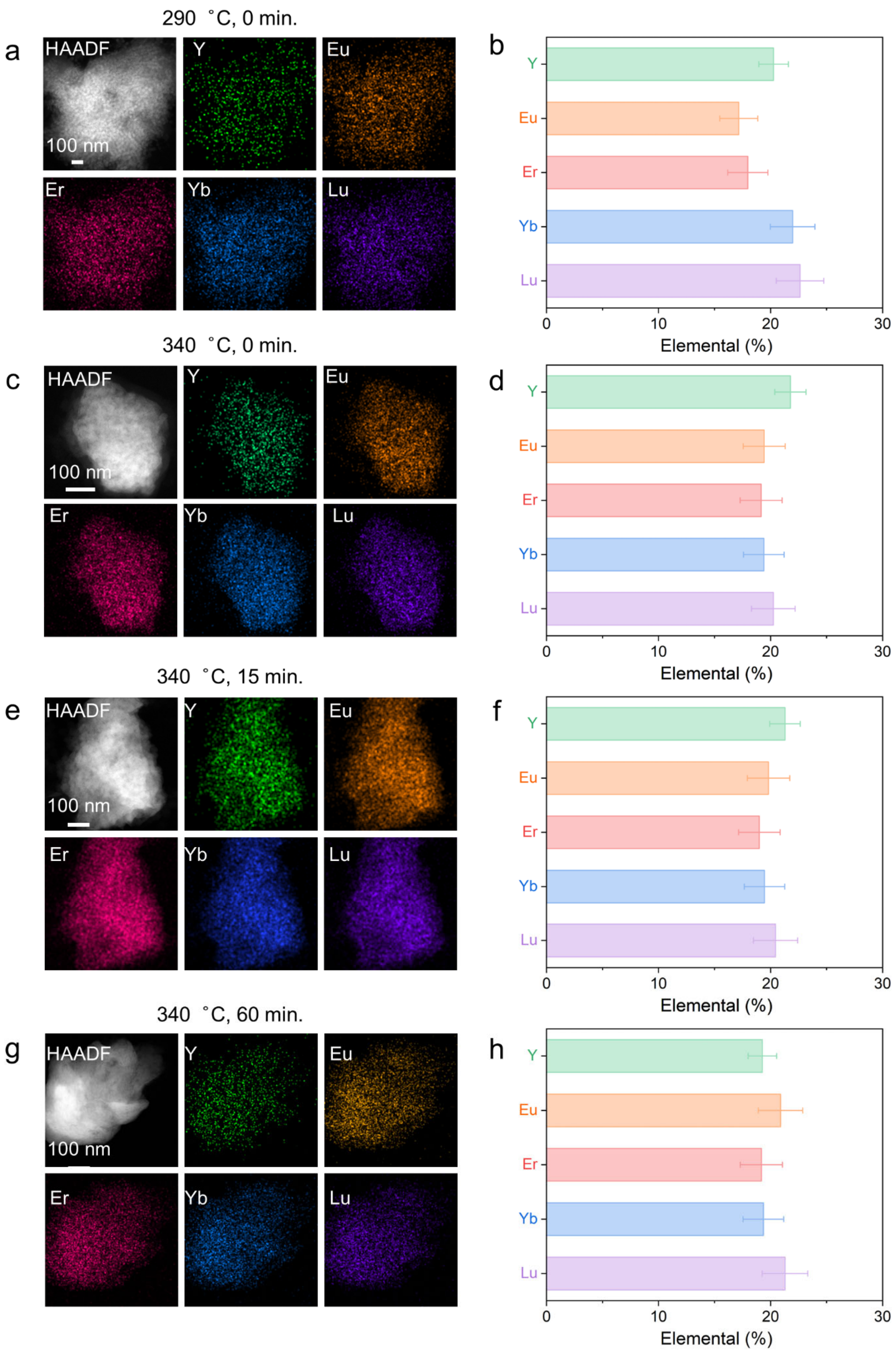

Figure S19. HAADF-STEM images, STEM-EDS elemental maps and corresponding elemental compositions of the temperature- and time-dependent aliquot samples collected during the synthesis of $(YEuErYbLu)_2O_3$ nanoplatelets.

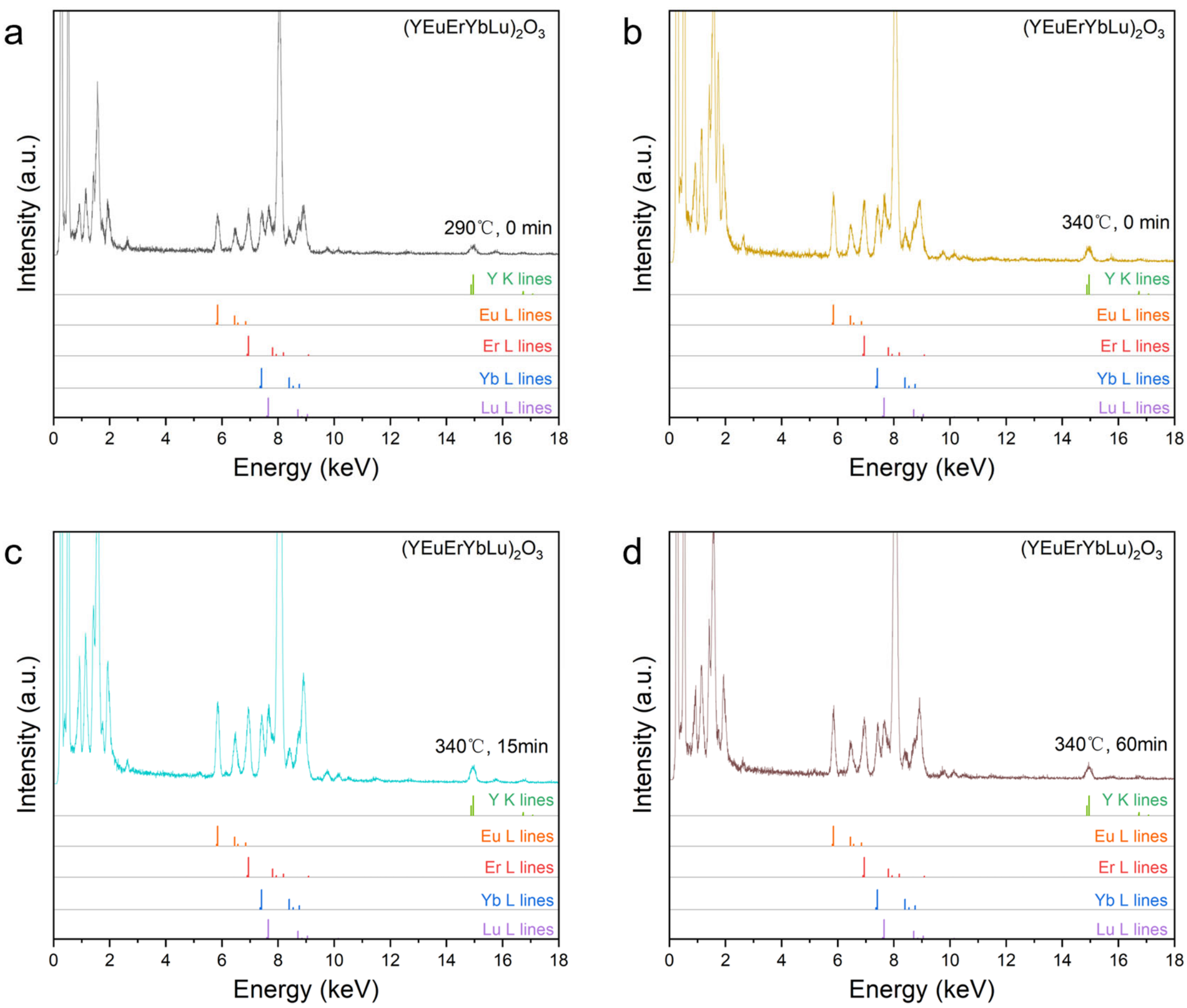

Figure S20. STEM-EDS spectra of aliquot samples collected during the synthesis of $(YEuErYbLu)_2O_3$ nanoplatelets at different reaction stages, with characteristic X-ray emission lines labeled for elemental identification: (a) 290 °C, 0 min; (b) 340 °C, 0 min; (c) 340 °C, 15 min; and (d) 340 °C, 60 min.

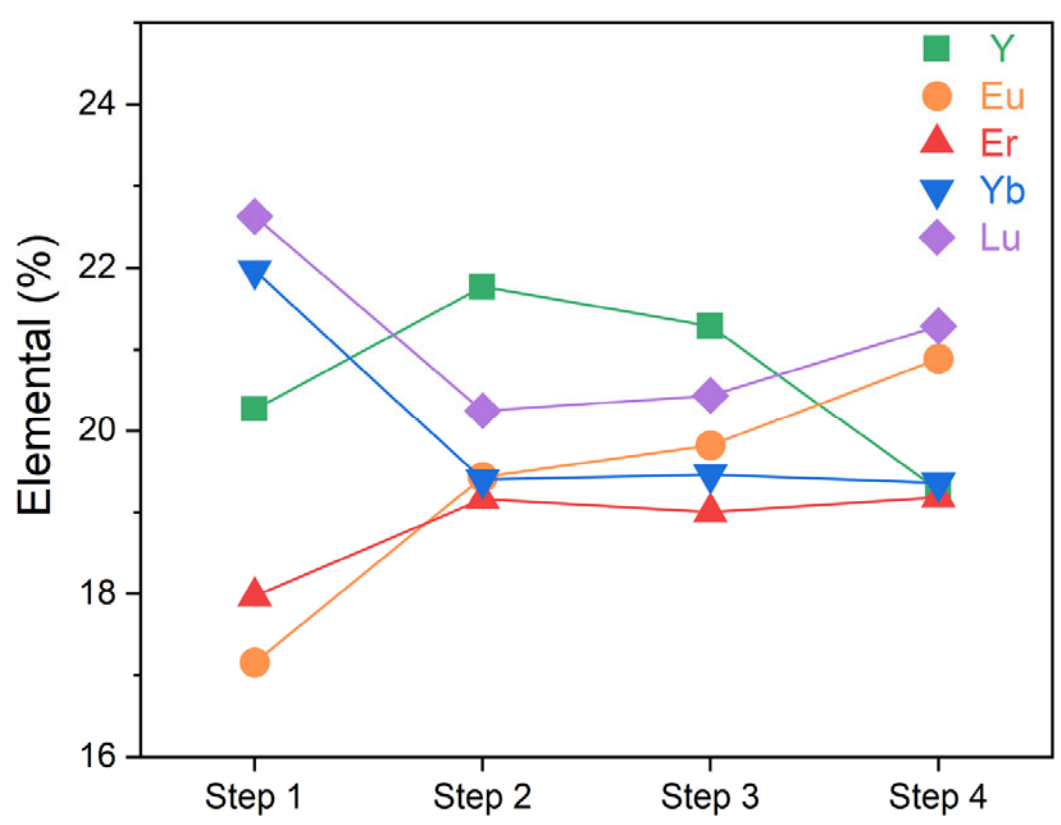

Figure S21. Elemental composition of the aliquot samples collected during the synthesis of $(YEuErYbLu)_2O_3$ nanoplatelets.

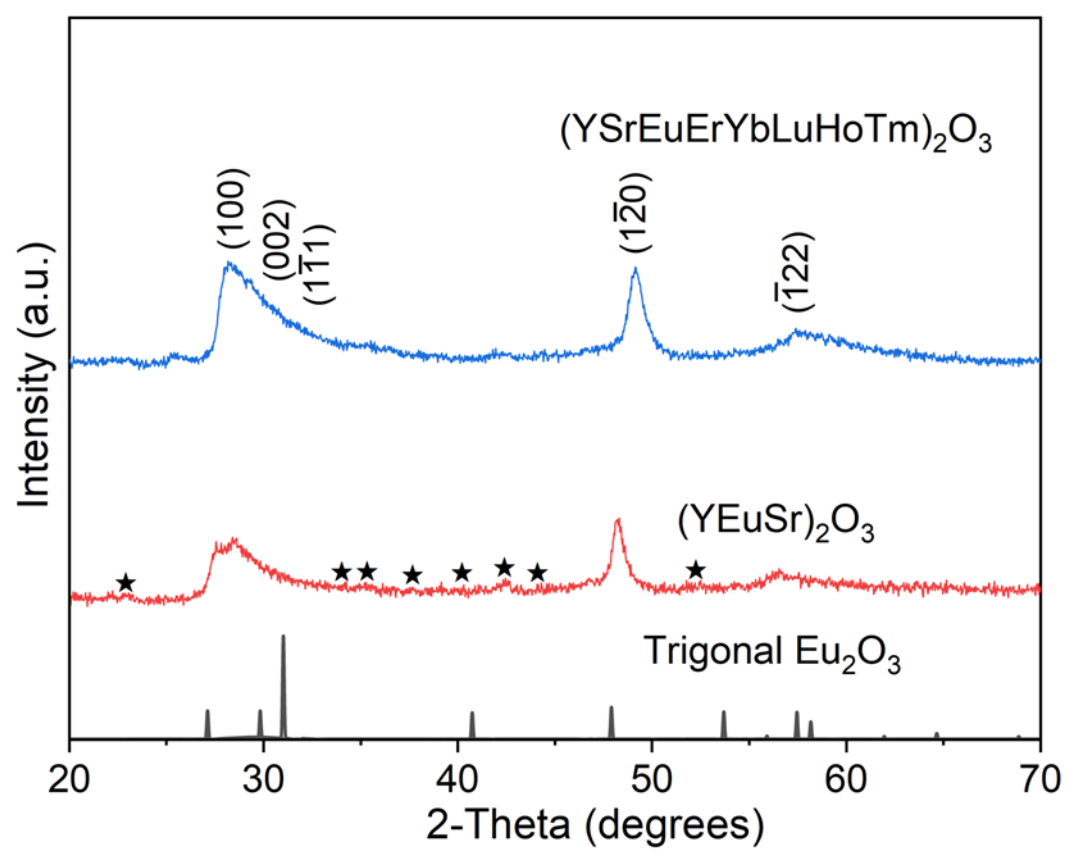

Figure S22. XRD patterns of low-entropy ternary $(YSrEu)_2O_3$ and high-entropy octonary $(YSrEuErYbLuHoTm)_2O_3$ nanoplatelets, with the reference pattern for bulk trigonal Eu_2O_3 shown for comparison.

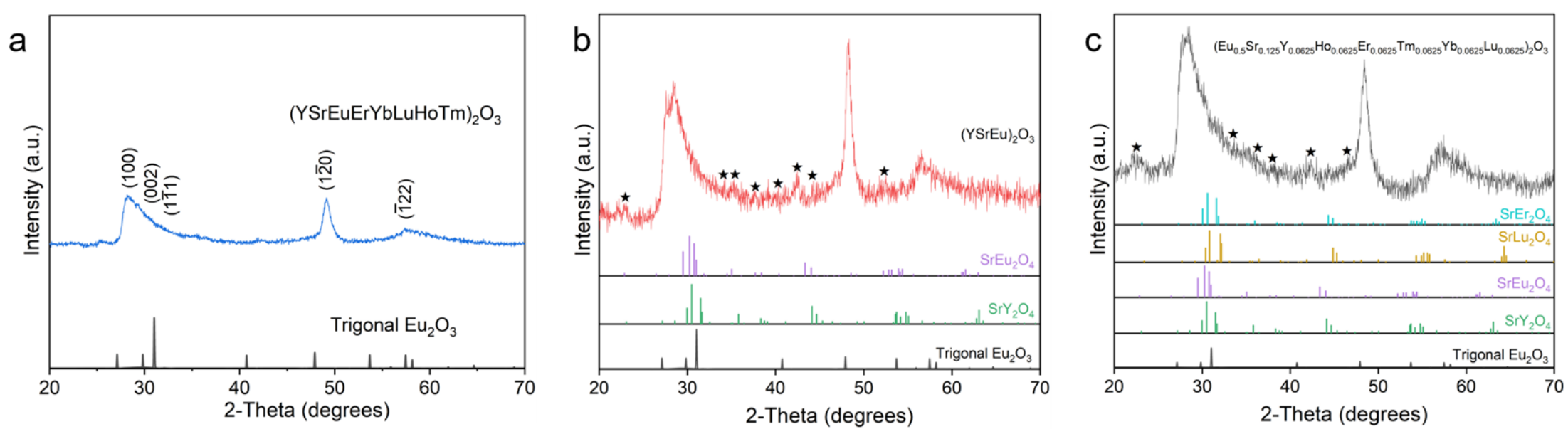

Figure S23. Enlarged XRD pattern of (a) $(YSrEuErYbLuHoTm)_2O_3$. (b) The ternary $(YSrEu)_2O_3$ sample together with the reference PDF patterns of trigonal Eu_2O_3, $SrEu_2O_4$ and SrY_2O_4 for comparison. The impurity peaks marked by asterisks can be assigned to the secondary phases of $SrEu_2O_4$ and SrY_2O_4. (c) The non-equimolar composition $(Eu_{0.5}Sr_{0.125}Y_{0.0625}Ho_{0.0625}Er_{0.0625}Tm_{0.0625}Yb_{0.0625}Lu_{0.0625})_2O_3$, in which the Sr content is fixed at 12.5 at.% while the configurational entropy is reduced.

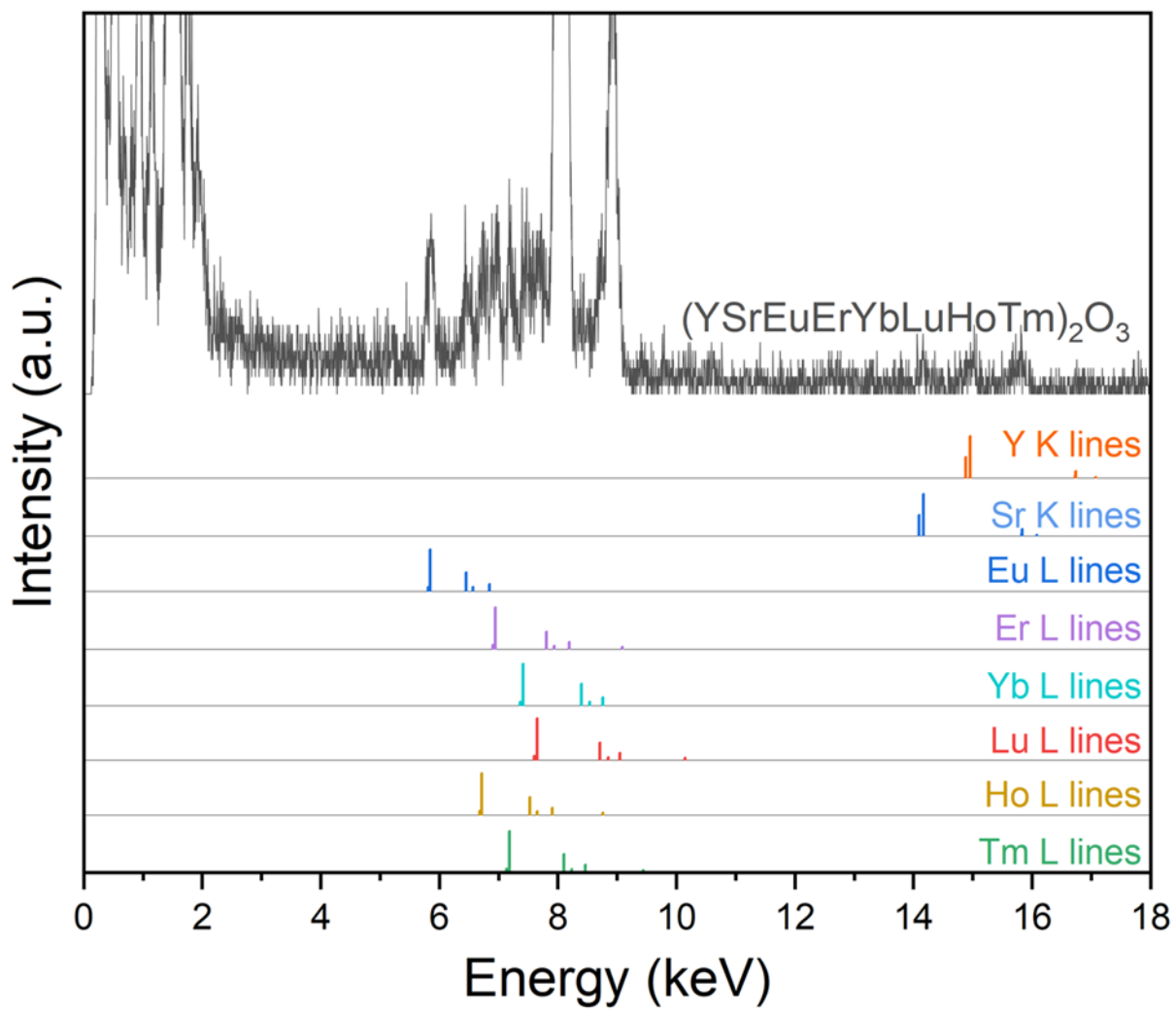

Figure 24. STEM-EDS spectrum of the octonary $(YSrEuErYbLuHoTm)_2O_3$ nanoplatelets, with characteristic X-ray emission lines labeled for elemental identification.

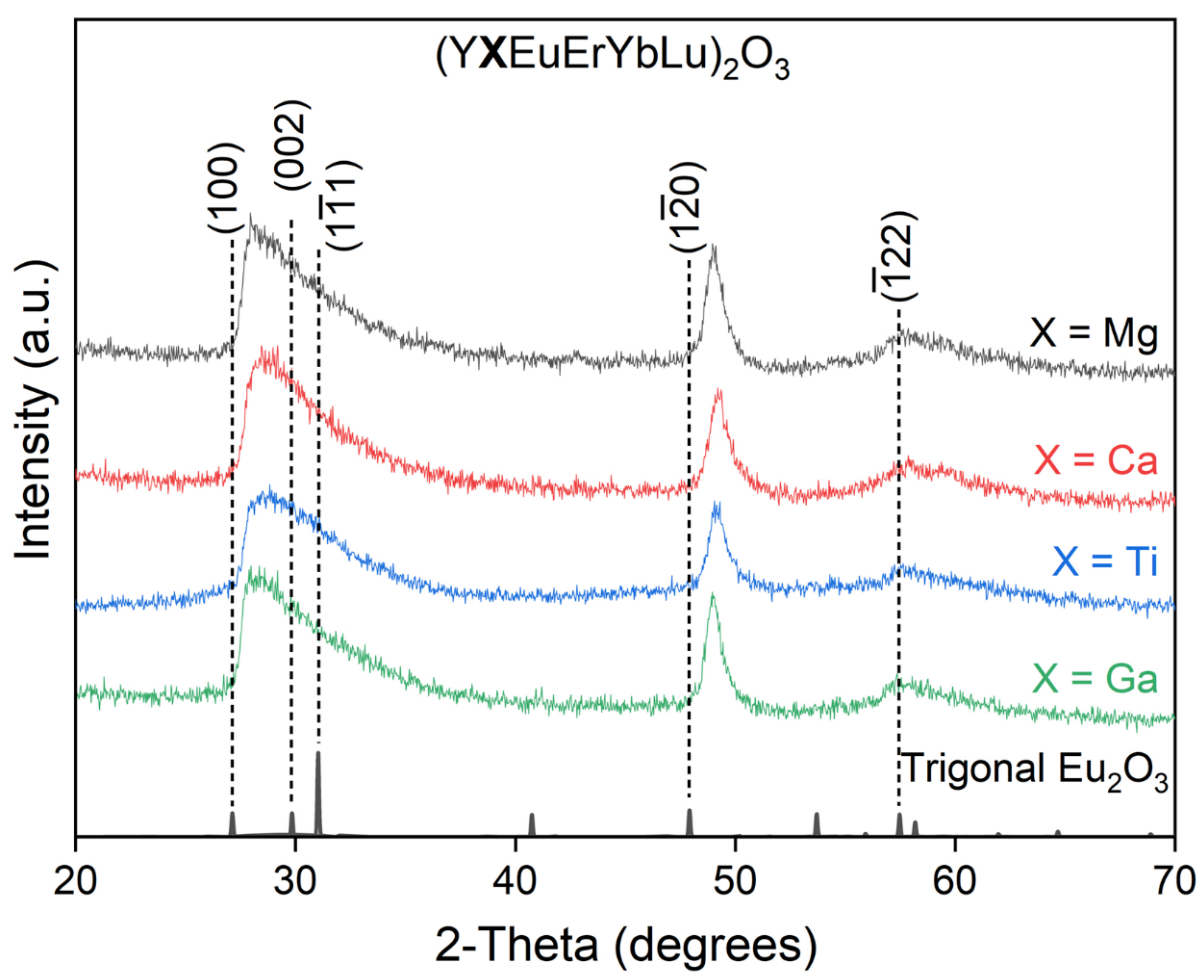

Figure S25. XRD patterns of Mg-, Ca-, Ti- and Ga-incorporated $(YEuErYbLu)_2O_3$ nanoplatelets.

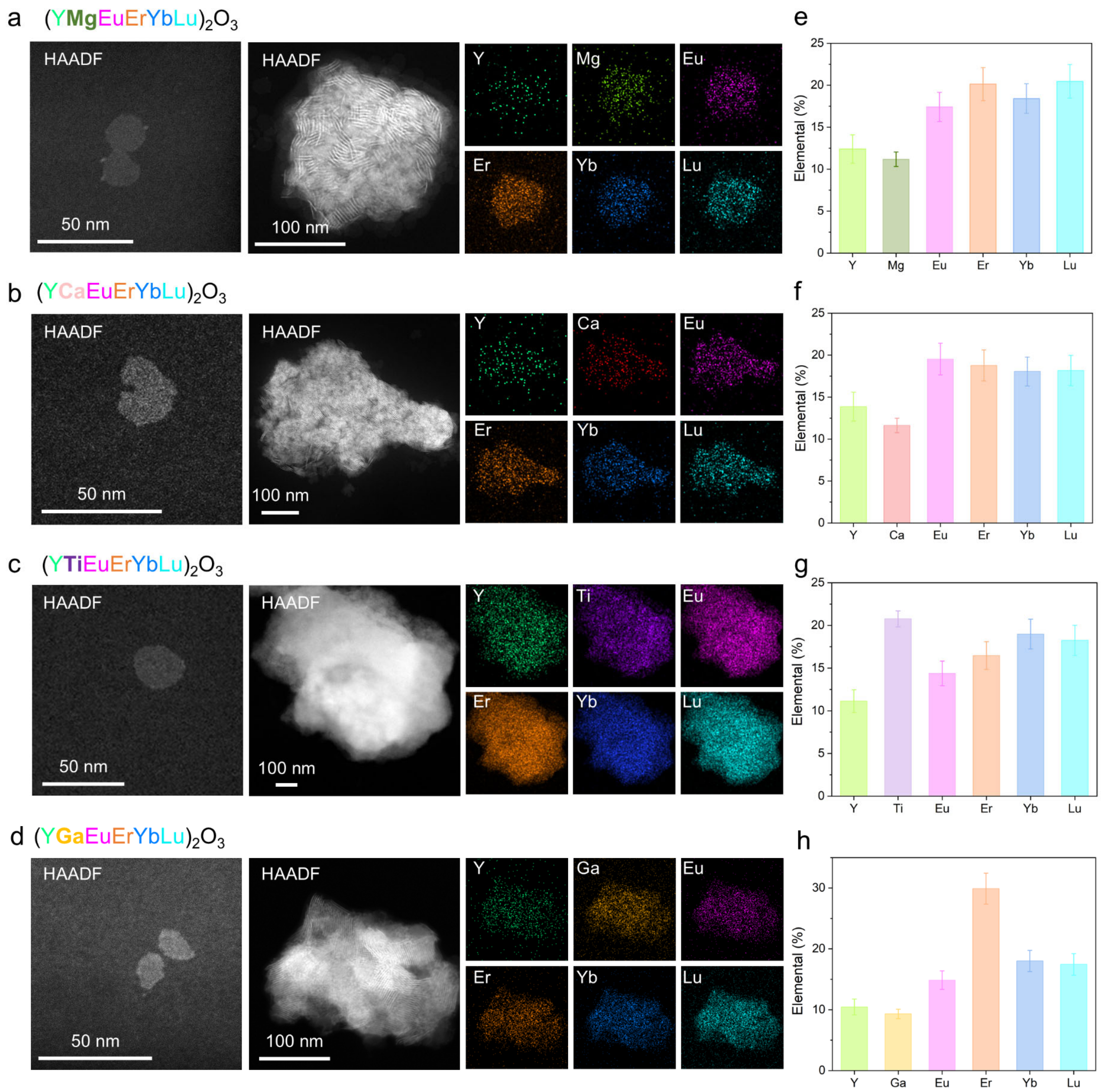

Figure S26. HAADF-STEM images, STEM-EDS elemental maps and elemental compositions of (a,e) Mg-, (b,f) Ca-, (c,g) Ti- and (d,h) Ga-incorporated (YEuErYbLu)2O3 nanoplatelets.

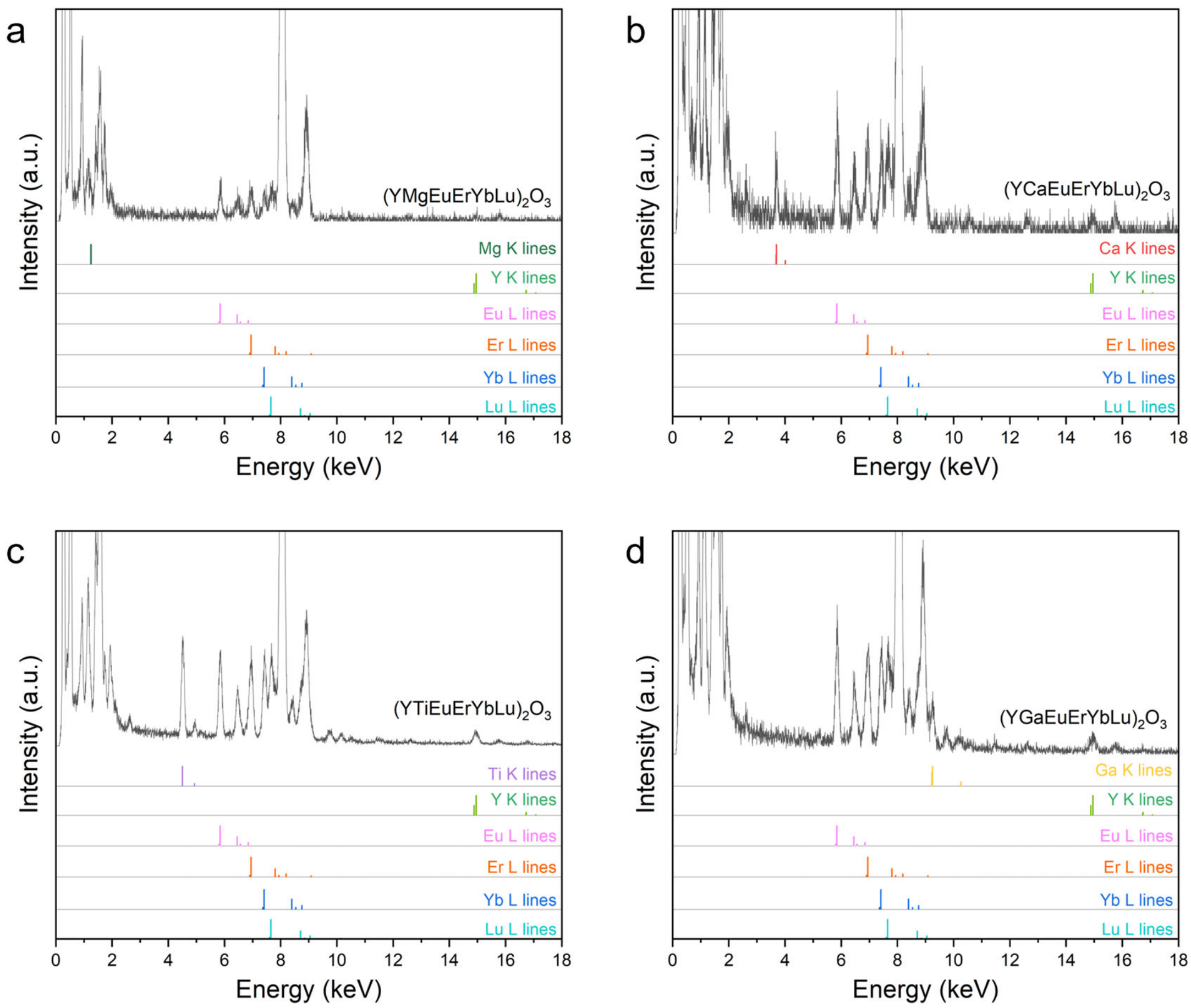

Figure S27. STEM-EDS spectra of quinary HE-REO nanoplatelets containing different non-rare-earth cations, with characteristic X-ray emission lines labeled for elemental identification: (a) $(YMgEuErYbLu)_2O_3$, (b) $(YCaEuErYbLu)_2O_3$, (c) $(YTiEuErYbLu)_2O_3$, and (d) $(YGaEuErYbLu)_2O_3$.

References

[1] Sun, Y.; Dai, S. High-entropy materials for catalysis: A new frontier. *Sci. Adv.* **2021**, *7*, eabg1600.

[2] Statham, P. J. Limitations to accuracy in extracting characteristic line intensities from X-ray spectra. *J. Res. Natl. Inst. Stand. Technol.* **2002**, *107*, 531–546.
